# *Vespa orientalis* pupae peptide hydrolysates modulate NF-κB signaling in LTA-induced pneumonia from clinical *Enterococcus faecalis* isolates: implications for gut microbiota

**DOI:** 10.3389/fnut.2025.1651499

**Published:** 2025-09-23

**Authors:** Mujeeb Ur Rahman, Muhammad Ilyas, Aamna Atta, Yamina Alioui, Sharafat Ali, Hidayat Ullah, Muhsin Ali, Ting Deng, Nabeel Ahmed Farooqui, Renzhen Ma, Mohammed Abusidu, Jiayi Wang, Liang Wang, Yi Xin

**Affiliations:** ^1^Department of Biotechnology, College of Basic Medical Science, Dalian Medical University, Dalian, China; ^2^Department of Biochemistry and Molecular Biology, College of Basic Medical Science, Dalian Medical University, Dalian, China; ^3^Guangdong Provincial Key Laboratory of Research and Development of Natural Drugs, and School of Pharmacy, Guangdong Medical University, Dongguan, China; ^4^Stem Cell Clinical Research Center, National Joint Engineering Laboratory, Regenerative Medicine Center, The First Affiliated Hospital of Dalian Medical University, Dalian, China

**Keywords:** gut microbiota, peptides hydrolysate, lipoteichoic acid, pneumonia, *Vespa orientalis*

## Abstract

**Introduction:**

Pneumonia continues to be a significant infectious disease, marked by ongoing lung inflammation, breathing difficulties, and high mortality rates, especially among young children in low-income nations.

**Methods:**

This study explored the therapeutic potential of peptide hydrolysates from *Vespa orientalis* pupae in a mouse model of *Enterococcus faecalis* lipoteichoic acid (LTA)-induced pneumonia. Mice were administered pupae peptide hydrolysate (PPH), and outcomes were evaluated based on clinical symptoms, histopathological analysis, cytokine profiling, expression of tight junction proteins, gut microbiota analysis via 16S rRNA sequencing, and NF-κB signaling activity in the lungs.

**Results:**

PPH treatment alleviated LTA-induced symptoms, reduced inflammation in the lungs and colon, and improved the balance of gut microbiota. It strengthens the intestinal barrier by increasing the levels of Mucin-2, Mucin-4, and tight junction proteins (Claudin-1, Occludin, ZO-1). Immune modulation was observed, with a decrease in pro-inflammatory cytokine levels, an increase in anti-inflammatory cytokine levels, and suppression of NF-κB signaling. Restoration of the gut microbiota composition further supports the therapeutic role of PPH.

**Discussion/conclusion:**

These results indicate that PPH mitigates LTA-induced pneumonia by reinforcing the intestinal barrier, modulating inflammatory pathways, including suppression of NF-κB, and restoring microbial balance. PPH represents a promising new approach for managing pneumonia as a chronic inflammatory disease.

## Graphical abstract

Schematic representation of *V. orientalis* pupae peptide hydrolysates on *Enterococcus faecalis* LTA-induced pneumonia. The figure depicts the experimental model of *Enterococcus faecalis*-derived lipoteichoic acid (LTA)-induced pneumonia in mice, subsequently treated with *V. orientalis* pupae peptide hydrolysates (PPH). PPH treatment facilitates microbiome restoration, as confirmed by 16S RNA analysis, resulting in a fortified gut barrier function while concurrently attenuating pulmonary inflammation. Mechanistically, bacterial products activate the TLR-2/MyD88 signaling axis, triggering IKKα/IKKβ-mediated phosphorylation of p65 and subsequent NF-κB nuclear translocation, ultimately modulating cytokine synthesis. The therapeutic outcomes include restoration of inflammatory cytokine homeostasis, repair of colonic and pulmonary parenchyma, inhibition of pathological cell signaling, microbiome revitalization, and metabolome reprogramming, which elucidate the gut-lung axis in pneumonia pathophysiology and treatment. Created in BioRender. Ur Rahman, M. (2025) https://BioRender.com. 
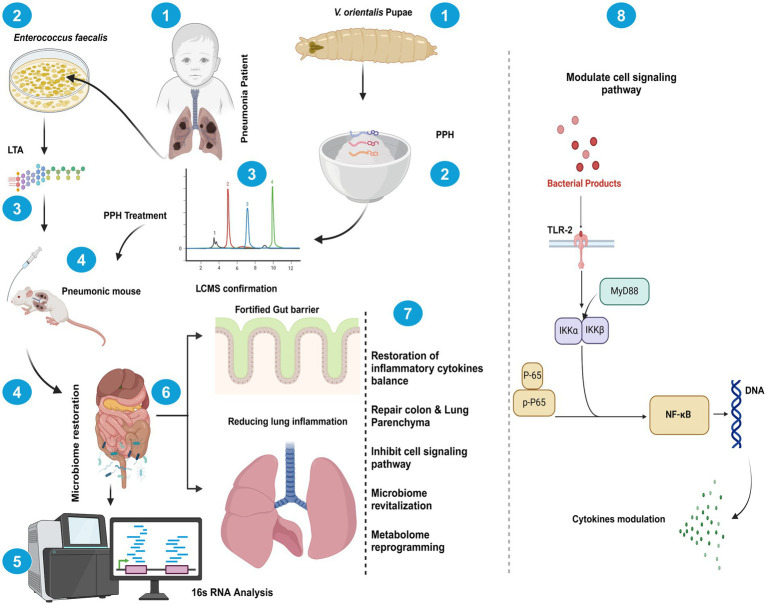

## 1 Introduction

Lower respiratory tract infections, particularly pneumonia, continue to pose a substantial global health challenge, significantly impacting morbidity and mortality rates worldwide. This issue is especially prevalent in developing regions, where inadequate healthcare infrastructure and limited therapeutic options exacerbate the situation. Bacterial pathogens play a significant role in this health crisis, and recent studies have elucidated the importance of the gut-lung axis in the pathogenesis of pneumonia (1). Discovered that *Enterococcus faecalis*, identified in pediatric pneumonia patients, is a crucial factor in promoting pulmonary inflammation through its virulent component, lipoteichoic acid (LTA).

Despite significant advancements that have been made, pneumonia treatment continues to rely solely on chemotherapy. The numerous side effects and lack of specific targeting of most current medications reduce their therapeutic effectiveness (2, 3). Consequently, there is an urgent need to discover novel agents with immunomodulatory, enhanced inhibitory effects, and selective targeting (3, 4). In recent years, targeted therapy has focused on finding natural and safe remedies to mitigate the side effects of drugs (5, 6). Extracts from natural products and medicinal plants have shown notable anti-inflammatory, antioxidant, and anticancer potential (4, 6–8).

Similarly, many bioactive compounds extracted from insects and mites have exhibited anticancer and immunogenic properties (9–11). Insects and their larvae are potentially rich sources of bioactive substances, though their potential has not been fully explored (12). Previous research has indicated that certain insect larvae, such as houseflies, possess anticancer, antioxidant, and antimicrobial qualities (10, 13, 14). Moreover, aqueous extracts and hydrolysates from insects like house crickets, grasshoppers, and silk moths contain beneficial bioactive peptides with anti-inflammatory and antioxidant activities (15, 16). Beyond these medicinal benefits, insects are a staple in the diets of at least two billion people worldwide, owing to their high protein and micronutrient content (17). Moreover, a study reported larvae of *Galleria mellonella* involves in polyethylene degradation (18). The consumption of antioxidant-rich foods aids in preventing diseases and cancer induced by oxidative stress (19). *Vespa orientalis*, commonly known as oriental hornets, are members of the *Vespidae* family. These insects are found in regions spanning the Middle East, Southwest Asia, Southern Europe, and Northeast Africa, where they form colonies (20). During winter, fertilized queens enter a state of hibernation (21, 22) and egg-laying occurs in autumn (23). The colony’s population reaches its peak in late summer and early fall (24). While their stings cause severe pain and potential allergic reactions in humans, certain Indian tribes utilize them to treat ailments such as colds and gastritis (22). These tribes also consume various *Vespa* species due to their high protein content (25).

Research has shown that the aqueous extract of the lesser banded hornet (*V. affinis*) exhibits antioxidant properties by activating antioxidant enzymes GST and CAT (26). The venom extracted from *V. orientalis* has demonstrated strong antimicrobial effects against numerous bacteria (27, 28) and has shown potential as an anticancer agent (29, 30). Furthermore, wasp venom contains biologically active peptides with anti-inflammatory, antimicrobial, and neuroactive properties. Recent studies have shown that analogs of venom-derived peptides, such as protonectin from *Parachartergus fraternus*, can modulate immune responses and reduce pro-inflammatory cytokines such as TNF-*α* (31). Another study in experimental rat models, wasp venom alleviated symptoms of rheumatoid arthritis, as demonstrated by enhanced histopathological outcomes and decreased levels of IL-6, TNF-α, IL-1β, and circulating inflammatory cells (32).

However, a major challenge in using insect venoms for treatment is their toxic effects on healthy cells (33). An alternative approach could involve the use of insect larvae and pupae. Nevertheless, there is currently a lack of research exploring the antioxidant, anticancer, and anti-inflammatory properties of *V. orientalis* larval extracts. Despite the wide array of pharmacological effects demonstrated by *V. orientalis* pupae, research has yet to explore the potential advantages of its peptide hydrolysate in addressing lipoteichoic acid (LTA)-induced inflammation in the lungs (1). This research seeks to examine the biological impact of these peptide hydrolysates on pulmonary inflammation. We propose that peptide hydrolysates extracted from *V. orientalis* pupae may mitigate LTA-induced lung inflammation, possibly offering a novel therapeutic approach for respiratory conditions associated with bacterial infections. Additionally, it examines the potential mechanism from the perspectives of intestinal and lung barrier repair, inflammatory factor reduction, and microbial composition changes in the colon of the BALB/c mouse model.

## 2 Materials and methods

### 2.1 Chemicals and reagents

In this study, a diverse array of materials and reagents were employed. Qingdao Yuanhaibo Biotechnology Co., Ltd. supplied the Brain Heart Infusion broth Medium and Enterococcus Agar medium. The stool DNA isolation kit was acquired from Chengdu Fuji Biotechnology Co., Ltd. Sinopharm Chemical Reagent Company provided trichloroacetic acid (TCA). 2X Taq Plus Master Mix II, HiScript III in one RT super mix perfect for RT-qPCR, and ChamQ Universal SYBR RT-qPCR Master Mix were procured from Nanjing Novozan Biotechnology Co., Ltd., Wuhan Sanying Biotechnology Co., Ltd., provided MUCIN-2 and MUCIN-4 rabbit polyclonal antibodies, while Proteintech, Wuhan, China. supplied Occludin, Claudin-1, and ZO-1. Severn Innovation Biotechnology Co, Ltd. (Beijing, China) provided an ultrasensitive ECL chemiluminescence detection kit. The additional reagents used in this investigation were of analytical grade and were obtained from authorized distributors.

### 2.2 Cultivation of *Enterococcus faecalis* and lipoteichoic acid (LTA) extraction

The *Enterococcus faecalis* strain employed in this study, designated as *E. faecalis* LTA-PP01 (Pediatric Pneumonia-01), was a clinical isolate obtained from the intestinal tract of a pediatric patient with pneumonia, with informed consent secured from the child’s guardians. The bacterial isolates were provided by the Microbiology Teaching and Research Department at Dalian Medical University. Its identification was confirmed through 16S rRNA gene sequencing. To ensure genetic stability and reproducibility, the strain has been preserved in 25% glycerol stocks at −80 °C. Lipoteichoic acid (LTA) was extracted from this virulent *E. faecalis* strain using a slightly modified version of the method described by (34). Briefly, *E. faecalis* was cultured in Brain Heart Infusion (BHI) broth at 37 °C for 16 h until reaching the logarithmic growth phase. The cells were subsequently harvested, washed three times with sterile PBS, and sonicated in 10 mL of sterile PBS (Toshiba Instruments Co., Tokyo, Japan). The bacterial suspension was mixed with a preheated 90% phenol solution at 65 °C, stirred for 30 min, cooled to 4 °C, and centrifuged at 3,000 × g for 10 min. The aqueous layer was extracted three times with sterile ultrapure water at 65 °C. To remove contaminating nucleic acids and proteins, DNase, RNase, and protease K (10 mg/mL; Solarbio, Beijing, China) were added and incubated for 45 min, followed by heat inactivation at 100 °C for 5 min. The aqueous phase was placed in a dialysis bag (molecular weight cutoff 15,000 Da; Solarbio, Beijing, China) and dialyzed against sterile water until no black precipitate was observed. The dialysate was then freeze-dried into a powder. The same batch of LTA was used in all experiments to ensure consistency, and the final product was stored at −20 °C until required.

### 2.3 Extraction of pupae peptides hydrolysate

The enzymatic hydrolysis method was used to harvest pupae peptide hydrolysate following a modified version of the procedure described in (35). The process was initiated by cleaning and grinding the pupae, which were then immersed in distilled water at 95 °C for 60 min using twice the volume of water. After soaking for an hour, the pupae remnants were strained through a 140-μm mesh and mixed with double the amount of distilled water. Bromelain was introduced at a 1% (w/w) ratio to facilitate the enzymatic breakdown of the pupae. The mixture was incubated at 50 °C for 7 h under continuous stirring. The enzymatic reaction was stopped by heating the solution to 100 °C for 20 min. The mixture was centrifuged at 14,000 x g for 20 min at 4 °C. The Bradford method was used to measure the concentration of the peptide hydrolysate. Finally, the supernatant containing the peptide hydrolysate was converted to a powder using a lyophilizer and stored at −20 °C for future use.

### 2.4 Distribution of molecular weights and amino acid profile of PPH

Peptide Profile Analysis by LC–MS/MS Peptide hydrolysate derived from *Vespa orientalis* pupae (PPH) was analyzed using an EASY-nLC 1,200 nano-LC system coupled with a Q Exactive HF Orbitrap mass spectrometer (Thermo Fisher Scientific). Sample preparation involved 10 kDa ultrafiltration, desalination using C18 SPE columns, freeze-drying, and reconstitution in 0.1% formic acid prior to analysis. Liquid chromatography was performed using a C18 PepMap column over a 120-min gradient. Mass spectrometry data were acquired in positive-ion mode employing data-dependent acquisition, with a full MS scan range of m/z 400–1800 at a resolution of 60,000, followed by MS/MS scans of the top 20 precursor ions. Data processing was performed using Proteome Discoverer 2.5. Peptide identification was achieved by using the UniProtKB database (*Vespa orientalis*, Taxonomy ID: 6760) with nonspecific enzyme cleavage. Mass tolerances were set at 10 ppm for precursor ions and 0.02 Da for-fragment ions. The fixed modification included carbamidomethylation (C), whereas the variable modifications comprised methionine oxidation and N-terminal acetylation. The false discovery rate (FDR) threshold for peptide identification was set at ≤1%. The non-hydrolyzed crude sample was not analyzed by LC–MS/MS because it would not yield identifiable peptides or contribute to peptide profiling.

### 2.5 Animal studies and protocol design

**Figure 1:**
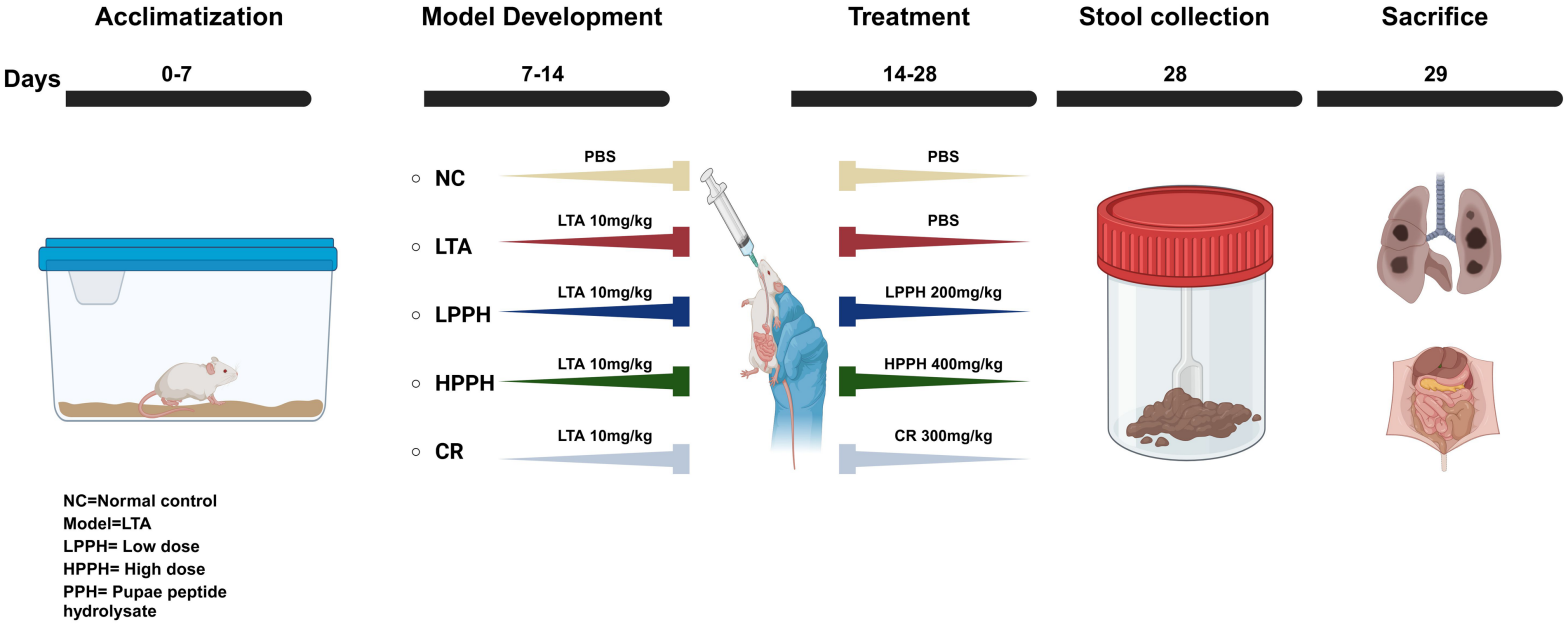
Illustrating experiment stages: acclimation, model development, and treatment. Control group (NC) received PBS orally, Model development: LTA, LPPH, HPPH, and CR groups received 10 mg/kg LTA orally for 1 week. From day 14, treatments included PBS for NC and LTA group, low/high PPH doses for LPPH/HPPH groups, and crude extract for CR. Fecal samples were collected on day 28, followed by mouse sacrifice.

Three-week-old male BALB/c mice, weighing 20–22 g, were obtained from Liaoning Changsheng Biotechnology Co. (Shenyang, China), which were chosen for their consistent health and minimal experimental variability. The mice were housed in a specific pathogen-free animal center (SPF level) at Dalian Medical University with free access to sterilized food and water. The animals were allowed to acclimatize at 25 °C for a minimum of 1 week before the experiments began. As shown in Figure 1, the mice underwent daily oral gavage treatments with LTA 10 mg/kg for a week, followed by treatment with pupae peptide hydrolysate (PPH). The mice were categorized into five groups: normal control (NC), lipoteichoic acid (LTA), low-dose PPH (LPPH), high-dose PPH (HPPH), and crude (CR). On the eighth day, the LPPH and HPPH groups were orally administered 200 and 400 mg/kg of PPH, respectively, while the crude group received 300 mg/kg of undigested pupae lysate. In dose-finding studies, the “low” and “high” doses of PPH, specifically 200 mg/kg and 400 mg/kg, were established based on the criteria of immune modulation, safety, and therapeutic efficacy within LTA-induced inflammatory models, considering the biological activity and tolerability in mice. The crude extract (CR) group administered 300 mg/kg of non-hydrolyzed *Vespa* orientalis pupae for comparison with enzymatically processed peptide hydrolysates. None of the administered doses resulted in weight loss, behavioral changes, or organ damage, confirming the safety of the selected concentrations. The NC and LTA groups administered an equivalent volume of phosphate-buffered saline (PBS) via oral gavage. All animal experiments were approved by the Animal Management and Use Committee of Dalian Medical University (approval number: 202410368). On day 28, fecal samples were collected prior to euthanasia. The mice were euthanized by intraperitoneal injection of sodium pentobarbital (200 mg/kg, 54 mg/mL working solution derived from a 325 mg/mL stock; injection volume 70–110 μL according to body weight 18–30 g). Death was confirmed by cervical dislocation following the cessation of respiration. All procedures complied with institutional guidelines and the AVMA Guidelines for the Euthanasia of Animals (2020). The colon, small intestine, spleen, and other organs were extracted, preserved in 4% formalin, and stored at −80 °C for subsequent analysis.

### 2.6 Measurement of body weight, disease activity index, and food and water intake

Body weight was recorded daily throughout the experimental duration. Additionally, food and water intake were assessed every third day (36–38). The severity of pulmonary inflammation in mice resulting from LTA was also assessed using various indicators. These include general behaviors (Activity Levels, Posture, Grooming), Breathing Patterns (Respiratory Rate; tachypnea-bradypnea, Effort, Nasal flaring, Open mouth breathing and Abdominal contractions), Respiratory Sounds (Wheezing or crackles), Physical Appearance (Nasal Discharge, Cyanosis; tail-ears-paws, Weight Loss), Response to Stimuli (Touch Response, Exploration Behavior), Fur and Skin (Fur Condition, Skin Turgor) (39, 40).

### 2.7 Histopathological analysis

Following the established sacrificial protocol, samples were meticulously extracted from the colon and lungs for subsequent analyses. To examine histological alterations, the collected tissues were sliced into 5 μm-thick sections using a microtome (Thermo Fisher Scientific, Waltham, MA, USA). These tissue sections were then subjected to hematoxylin–eosin (HE) staining, adhering to the procedure described by Fischer et al. (41). Initially, tissue sections were deparaffinized in fresh xylene for 10 min. Subsequently, the sections were rehydrated using a series of ethanol concentrations to restore their water content. Hematoxylin–eosin staining was performed on the tissue sections. After preparation, the slides were examined using a Leica Microsystems microscope (Wetzlar, Germany). The analysis and evaluation of histological morphology, tissue regeneration, and inflammation were conducted employing a scoring system delineated in Table 1 (42, 43).

**Table 1:** Histological evaluation of colon and lung tissue samples, assessment of inflammation and regeneration using scoring.

Feature	Score	Description
Regeneration	4	No tissue repair
	3	Surface epithelium not intact
	2	Regeneration with crypt depletion
	1	Almost complete regeneration
	0	Complete regeneration or normal tissue
Inflammation	3	Severe
	2	Moderate
	1	Slight
	0	None

### 2.8 Immunohistochemical staining for mucus in colon and lungs

Immunohistochemistry (IHC) analysis was employed to examine Mucin-2 and Mucin-4 expressions in colon and lung tissue samples. The process began with deparaffinization of the tissue slides using xylene, followed by rehydration through an ethanol gradient. Antigen retrieval was achieved by heating the slides in citrate buffer (10 mM, pH 6.0) using a microwave. After cooling, the slides underwent three 5-min washes in PBS (pH 7.4). To block endogenous peroxidase activity, the sections were incubated in H2O2 solution (Sangon Biotech Co. Ltd., Shanghai, China) for 25 min at room temperature in the dark and then blocked with 5% BSA in PBS. The staining procedure followed the manufacturer’s guidelines (SP-KIT9720 Immunohistochemistry Staining Kit; MXB Biotechnologies, Beijing, China). Primary antibodies anti-Mucin-2 (Proteintech, 27,675-1-AP) and anti-Mucin-4 were applied at a 1:1,000 dilution and incubated overnight at 4 °C. The slides were then washed three times with PBS for 10 min each, before incubation with secondary antibody for 1 h at room temperature. Following another wash, 3,3′-diaminobenzidine (DAB) was applied for 5 min, then rinsed under running tap water for 10 min. Hematoxylin counterstaining was performed for 5 min, followed by another 10-min tap water rinse. The slides were dehydrated through an ethanol series, cleared with xylene, and mounted using neutral balsam (cat-G8590, Solarbio). Immunolabeled cells were evaluated by observing three random fields per slide under a microscope. Semi-quantitative analysis was performed using ImageJ software.

### 2.9 Periodic acid-Schiff and Alcian blue

Periodic acid-Schiff (PAS) staining was employed to assess neutral mucin, mucus epithelium thickness, and goblet cells in the colon. The procedure commenced with xylene-based deparaffinization of tissue slides, followed by rehydration using a series of ethanol solutions with progressively decreasing concentrations. After rehydration, the periodic acid reagent was applied for 5 min at room temperature, followed by three ultra-filtered water rinses. Schiff reagent was then utilized for 10 min in a sealed container, and the slides underwent an 8-min wash under running water. Hematoxylin counterstaining and a subsequent 7-min tap water rinse were performed. The slides were then dehydrated in ethanol, rendered transparent with xylene, and mounted using neutral balsam (cat-G8590; Solarbio). To specifically detect epithelial acid mucins and non-sulfated and sulfated acid mucins, Alcian blue staining (AB) at pH 2.5 was utilized. Following deparaffinization and hydration, the slides were immersed in 1% aqueous alizarin blue acetate solution for 10 min and then washed thrice for 6 min each. Oxidation was achieved by immersing in 1% periodic acid solution for 5 min, followed by two 6-min ultra-pure water washes. The slides were then stained with Schiff’s solution for 10 min and washed with tap water for 10 min. Dehydration was performed using increasing alcohol concentrations. Finally, the slides were dried and mounted to complete the process.

### 2.10 Immunofluorescent staining for Claudin-1, Occludin and ZO-1

Immunofluorescent staining was used to assess Claudin-1, Occludin, and ZO-1 expression levels in the colon tissue of mice from various experimental groups. A paraffin-embedded colon tissue section, 5 μm thick, was placed on a slide for analysis. The slides underwent deparaffinization in xylene twice for 10 min each, followed by rehydration using a descending ethanol concentration gradient. Antigen retrieval was performed by subjecting the slides to citrate buffer in a microwave oven. The tissue section was subsequently blocked with reagents for 20 min to prevent non-specific binding. After rinsing with PBS, the slides were incubated overnight at 4 °C with primary antibodies against Claudin-1, Occludin, and ZO-1. Following additional washes, the slides were exposed to secondary antibodies conjugated with fluorescein isothiocyanate (FITC) (Proteintech, Wuhan, China) for 1 h at room temperature under dark conditions. To visualize cell nuclei, 4′,6-diamidino-2-phenylindole (DAPI) staining was conducted for 5 min, followed by three 5-min PBS washes. Slides were mounted and examined under a microscope.

### 2.11 Measurement of serum cytokine levels

Blood samples were obtained immediately following euthanasia and subjected to centrifugation at 6,000 × g for 15 min to isolate serum. The serum samples were subsequently stored at −80 °C for future analysis. Quantification of pro-inflammatory and anti-inflammatory cytokines in the serum samples was performed using ELISA kits (Shanghai Jianglai Industrial Ltd.) in accordance with the manufacturer’s protocol.

### 2.12 Quantification of mRNA levels via real-time PCR

Total RNA was extracted from colon and lung tissue samples using Triazole reagents from Thermo Fisher Scientific and stored at −80 °C. The levels of messenger RNA (mRNA) in these tissues were measured using reverse transcription-quantitative polymerase chain reaction (RT-qPCR). Complementary DNA (cDNA) was synthesized using a reverse transcriptase kit, and RT-qPCR was performed using the SYBR Green RT-qPCR kit from Takara (Japan). Sangon Biotech Shanghai, China, produced all primers used in this study are listed in.

Table 2 each sample was analyzed in triplicate, and relative gene expression was determined and evaluated using the Line Gene 9,660 system software. GraphPad Prism was used to assess differences between various experimental groups.

**Table 2:** List of primers used in QPCR to check gene expression of mRNA level.

Type	Forward (5΄–3΄)	Reverse (3΄–5΄)
TNF-α	CATCTTCTCAAAATTCGAGTGACAA	TGGGAGTAGACAAGGTACAACCC
IL-1β	TGGACCTTCCAGGATGAGGACA	GTTCATCTCGGAGCCTGTAGTG
IL-17	GACTCTCCACCGCAATGAAGAC	CTCTTCAGGACCAGGATCTCTTG
TGF-β	CATTGCTGGTCCAGTCTGCTTCG	TGGTGAATGACAGTGCGGTTATGG
IL-10	CGGGAAGACAATAACTGCACCC	CGGTTAGCAGTATGTTGTCCAGC
β-actin	ATCGCTGCGCTGGTCG	GTCCTTCTGACCCATTCCC

### 2.13 Modulation of PPH on inflammatory signaling pathways and TJS expression

Protein extraction was performed using RIPA lysis buffer. The extracted proteins were then separated by SDS-PAGE on 8 to 12% gradient gels. Following separation, the proteins were transferred onto a PVDF membrane for immunodetection analysis. The membrane was first blocked with 5% skim milk for 2 h and then thoroughly washed with TBST to eliminate excess blocking agents and contaminants. The membrane was then incubated with the primary antibody overnight at 4 °C. After this step, the membrane was washed extensively to remove the unbound primary antibody. The membrane was then exposed to a secondary antibody linked to the appropriate detection moiety for 1 h at room temperature. Additional washing steps were carried out to remove excess secondary antibodies and any remaining contaminants. Finally, protein bands were visualized using an ECL substrate, and imaging was performed with a gel documentation system.

### 2.14 16S rRNA gene sequencing and analysis

Genomic DNA was extracted from fecal samples using a commercial stool DNA extraction kit (PowerMax DNA Isolation Kit from MoBio Laboratories). The V3–V4 hypervariable regions of the 16S rRNA gene were amplified using the primers 341f (CCTACGGGAGGCAGCAG) and 518r (ATTACGCGGCTGCTGG), followed by sequencing on the Illumina NovaSeq platform. The raw sequencing data were processed using QIIME2 (v2022.2), where quality filtering, denoising, chimera removal, and ASV generation were executed using the DADA2 plugin. ASVs were clustered into OTUs at a 97% similarity threshold, and taxonomy was assigned using the SILVA 138 database via a Naive Bayes classifier. Rarefaction was applied to normalize the data based on the minimum sequencing depth. Alpha diversity metrics, including Shannon and Chao1, and beta diversity, using Bray–Curtis, were calculated, and PCoA was employed to visualize beta diversity. Functional profiling of the microbial communities was conducted using PICRUSt2, which predicted KEGG orthologs (KOs) and pathway-level functions. Differential abundance of functions was analyzed using STAMP, and final figures were generated using GraphPad Prism 9.0.

### 2.15 Statistical analysis

The experiment was conducted in triplicate to ensure reliability. Statistical analyses were performed using Prism 10 (GraphPad Software, San Diego, CA, USA). For comparison of multiple groups, one-way analysis of variance was employed, with a *p*-value below 0.05 considered statistically significant.

## 3 Results

### 3.1 Identification and analysis of peptide

**Figure 2:**
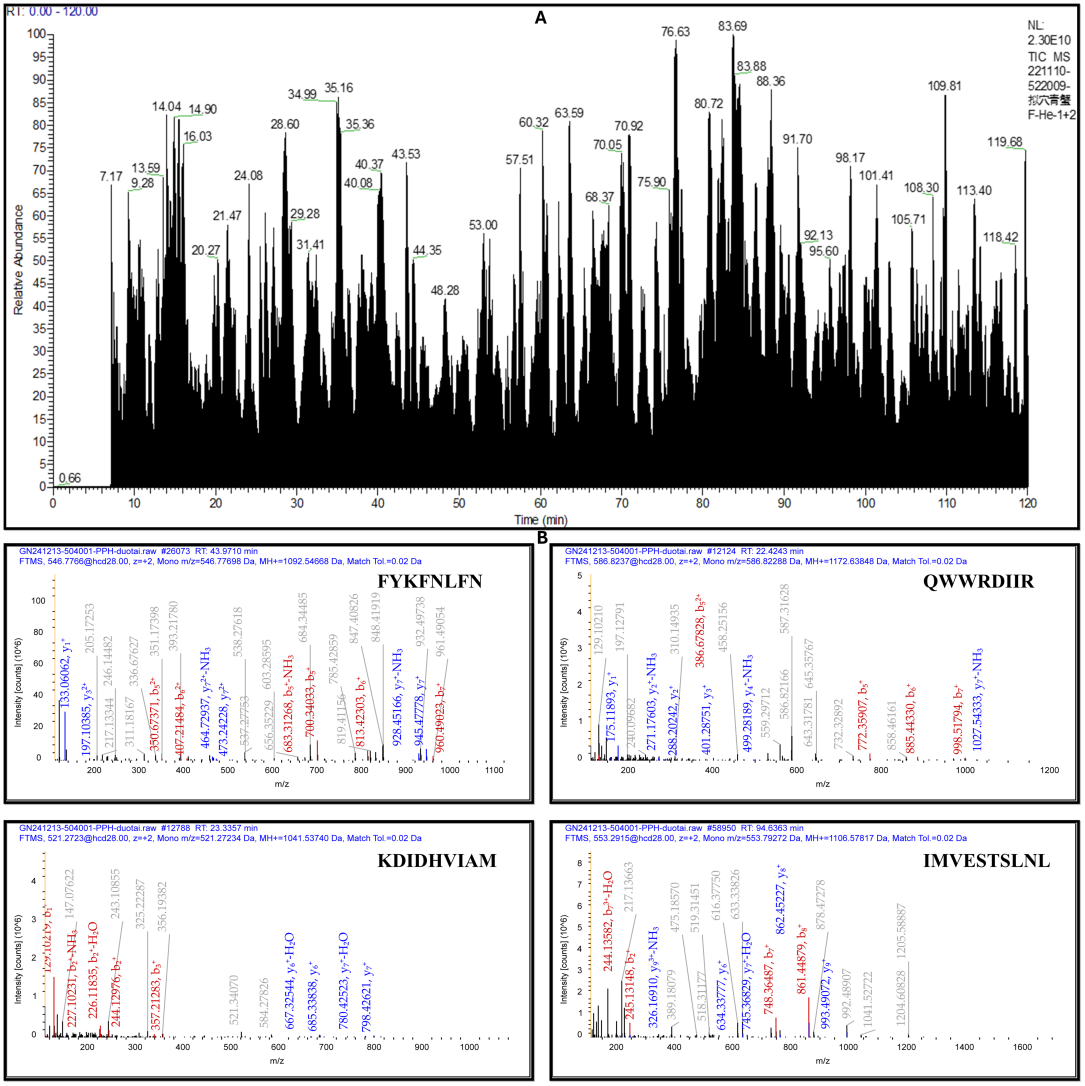
MALDI-TOF-MS analysis of peptides from *Vespa orientalis* pupae peptide hydrolysate. **(A)** The mass spectrum shows distinct peaks at specific m/z values, **(B)** representing peptides with unique molecular masses and varying amino acid compositions, indicating the heterogeneous nature of the hydrolysate.

Following enzymatic digestion of the pupae peptide hydrolysate with bromelain, the sample underwent lyophilization and subsequent mass spectrometric analysis. The mass spectrometry results revealed 12 distinct peaks, each corresponding to a peptide with a unique molecular weight and an amino acid sequence, as illustrated in the accompanying (Figure 2A and Table 3), along with Supplementary Table 1. High-resolution mass spectra obtained via liquid chromatography-mass spectrometry (LC–MS) analysis elucidated the fragmentation patterns of these peptides. The spectra exhibited charged precursor ions (z = +), with the observed m/z values corresponding to the monoisotopic measurements. The calculated monoprotonated molecular weights (MH+) were consistent with peptides comprising approximately 12–22 amino acid residues, depending on their specific composition, as shown in Figure 2B, along with Supplementary Figure 1. This detailed characterization provides valuable insights into the hydrolysate composition and successful digestion of pupal proteins into smaller bioactive components.

**Table 3:** Peptides identified from the hydrolysate exhibited varying molecular masses and distinct amino acid compositions.

S.No	UniProt accession No.	Peptide sequence	Length	Mol. weight (kDa)	Protein name	Function
1	A0A6B9QN21	FYKFNLFN	8	43.7	Apolipophorin-III-like	Lipid transport, immune response
2	A0A343B6T9	QWWRDIIR	8	31.1	Heat shock 70 kDa protein	Molecular chaperone, stress response
3	A4UA14	KDIDHVIAM	9	88.4	Vitellogenin	Antioxidant, antimicrobial function
4	A0A8F7CIX6	IMVESTSLNL	10	24.7	Phospholipase A2	Inflammatory modulation
5	A0A384ZVN2	KYDPTGEYKE	10	14.2	Chemokine-like protein	Immune signaling
6	A0A384ZVN2	DNIDVDQILK	10	14.2	Chemokine-like protein	Immune signaling
7	W8TKD9	VTIIDAPGHR	10	19.0	Serine protease inhibitor	Anti-inflammatory regulation
8	E9RHR1	LNPQIDTLTFQ	11	126.3	Cuticle protein	Structural function, barrier integrity
9	C0HLL3	KSDLSSTQVVF	11	33.9	Peroxiredoxin	Antioxidant, immune modulation
10	A0A384ZVN2	YDNIDVDQILK	11	14.2	Chemokine-like protein	Immune signaling
11	A0A1S5YCI0	KNINRFFLLVIMF	13	60.1	Allergen-like protein	Potential immunoreactivity
12	A0A384ZVN2	TKYDNIDVDQILK	13	14.2	Chemokine-like protein	Immune signaling

### 3.2 PPH alleviates clinical symptoms in LTA-induced pneumonia mice

**Figure 3:**
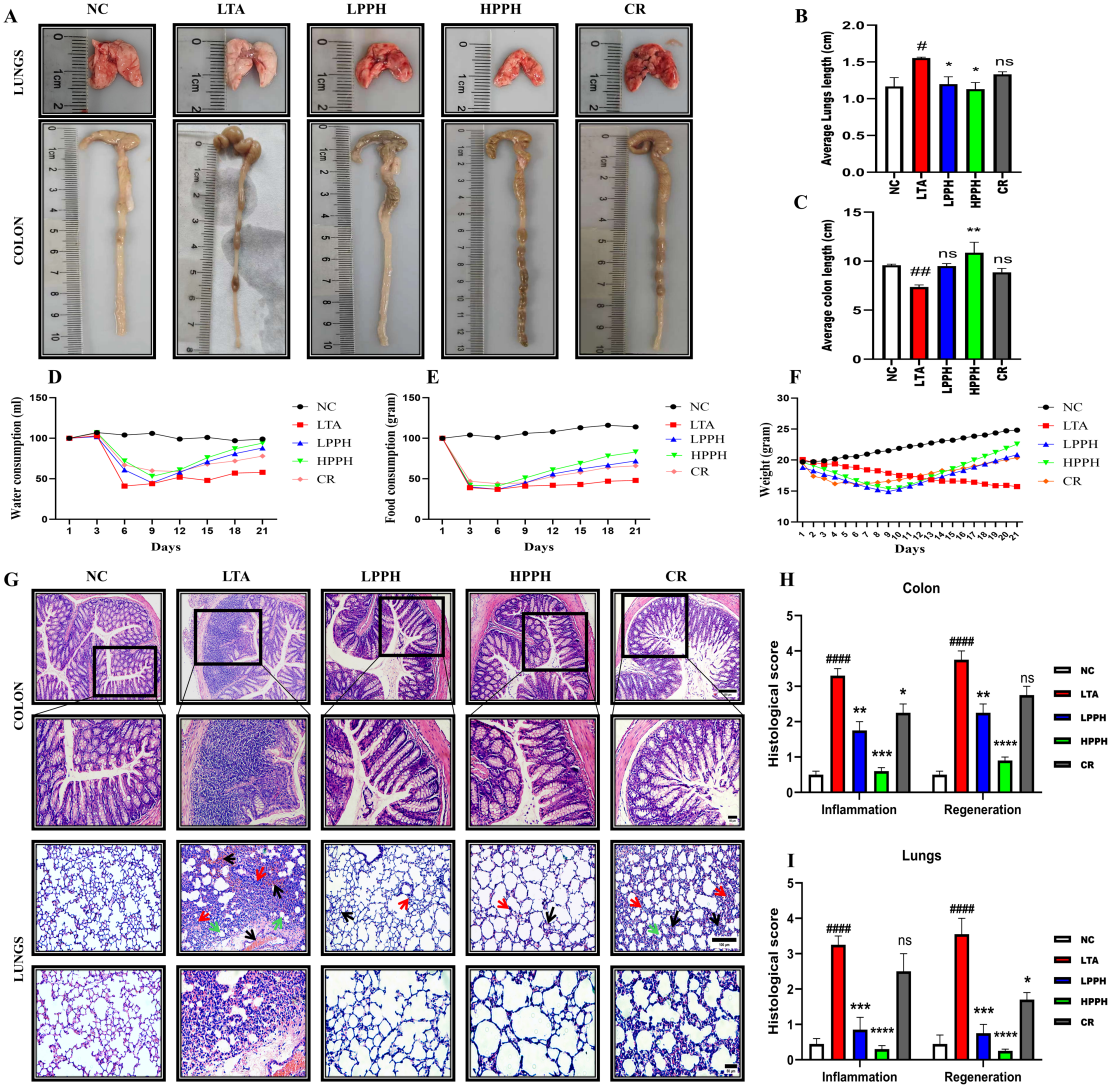
Effects of treatments on pneumonia progression, organ characteristics, and histopathological analysis. **(A)** Representative macroscopic images of lungs and colons from different treatment groups; **(B,C)** Organ index measurements for lungs and colons; **(D–F)** Physiological parameters: water intake, food consumption and body weight changes over the experimental period; **(G)** Histopathological H&E staining showing colon (upper panels) and lung tissues (lower panels) with alveolar structure and inflammatory infiltration (10×, 20 × magnification; scale bars: 100 and 50 μm); **(H,I)** Histological scoring for inflammation and regeneration parameters in colon and lung tissues. Groups: NC (Normal Control), LTA (Lipoteichoic Acid-induced pneumonia), LPPH (Low-dose PPH), HPPH (High-dose PPH), CR (Crude extract). *p < 0.05, **p < 0.01, ***p < 0.001, ****p < 0.0001 vs. LTA group; #*p* < 0.05, ##*p* < 0.01, ###*p* < 0.001 vs. NC group, ####*p* < 0.0001; vs. NC group; ns: not significant. Data presented as mean ± SEM.

The efficacy of PPH therapy was examined in mice with LTA-induced pneumonia following a 14-day treatment course. Various Disease Activity Index (DAI) metrics were assessed, including fur and skin deterioration, body temperature elevation, general behavior, breathing patterns, respiratory sounds, stimulus response, physical appearance, and dark stool presence (39, 40, 44). As shown in Figure 3A, representative macroscopic images revealed that the LTA group displayed significantly inflamed lungs and shortened colons compared to the negative control. Figures 3B,C demonstrate quantitative analysis showing that LPPH and HPPH treatment resulted in substantial improvement in average lung and colon lengths, whereas CR did not yield significant increases.

Furthermore, the impact of LTA on physiological parameters was evident in Figures 3D–F, showing reduced water intake, food consumption, and significant body weight loss in the LTA group. However, PPH administration enhanced water intake and food consumption while promoting notable weight recovery after 2 weeks in a dose-dependent manner, with LPPH and HPPH demonstrating greater improvement compared to CR, confirming the protective effects of PPH treatment.

### 3.3 PPH improves the histomorphology of the colon and lungs

To assess the effects of PPH treatment in mice with LTA-induced pneumonia, hematoxylin and eosin (HE) staining was used to count goblet cells and examine the histological structure of the colon and lungs. The results showed that the normal NC group of mice had a colon with clearly defined, closely arranged columnar epithelium and distinct mucosal and submucosal layer boundaries. Inflammation was minimal, and goblet cells were abundant. In contrast, the LTA-treated group exhibited significant abnormalities, including extensive tissue damage, inflammation, altered and shallow crypt structures, decreased goblet cell numbers, and disrupted tissue organization. However, PPH administration caused dose-dependent improvements in the damaged colon tissue. The CR group demonstrated moderate enhancement with reduced inflammation and tissue damage compared to the LTA group. The LPPH and HPPH treatment groups showed substantial improvement, with a near-normal histological structure, minimal inflammation, and restored tissue integrity, as shown in Figure 3G. Additionally, the LTA group exhibited lung disruption, characterized by collapsed alveolar cells, merged lung septa, and the presence of inflammatory cells, accompanied by significant neutrophil infiltration, as depicted in Figure 3G. Lung hemorrhage is indicated by a black arrow, alveolar wall thickening by a red arrow, and debris affected by a green arrow (45). These observations suggested that the lungs of mice in the LTA group suffered severe damage and inflammatory changes (*p* < 0.05). Furthermore, PPH administration demonstrated a therapeutic effect by improving lung and intestinal morphology and specifically restoring the defined margin, as illustrated in Figure 3G. Quantitative histological scores for colon and lung tissue inflammation and regeneration parameters, as shown in Figures 3H,I, respectively, HPPH exhibited a more pronounced effect than LPPH>CR when compared to the model LTA group in reducing inflammation and promoting regeneration in the colon and lungs, respectively.

### 3.4 Periodic acid–Schiff (PAS) and Alcian blue (AB) staining

**Figure 4:**
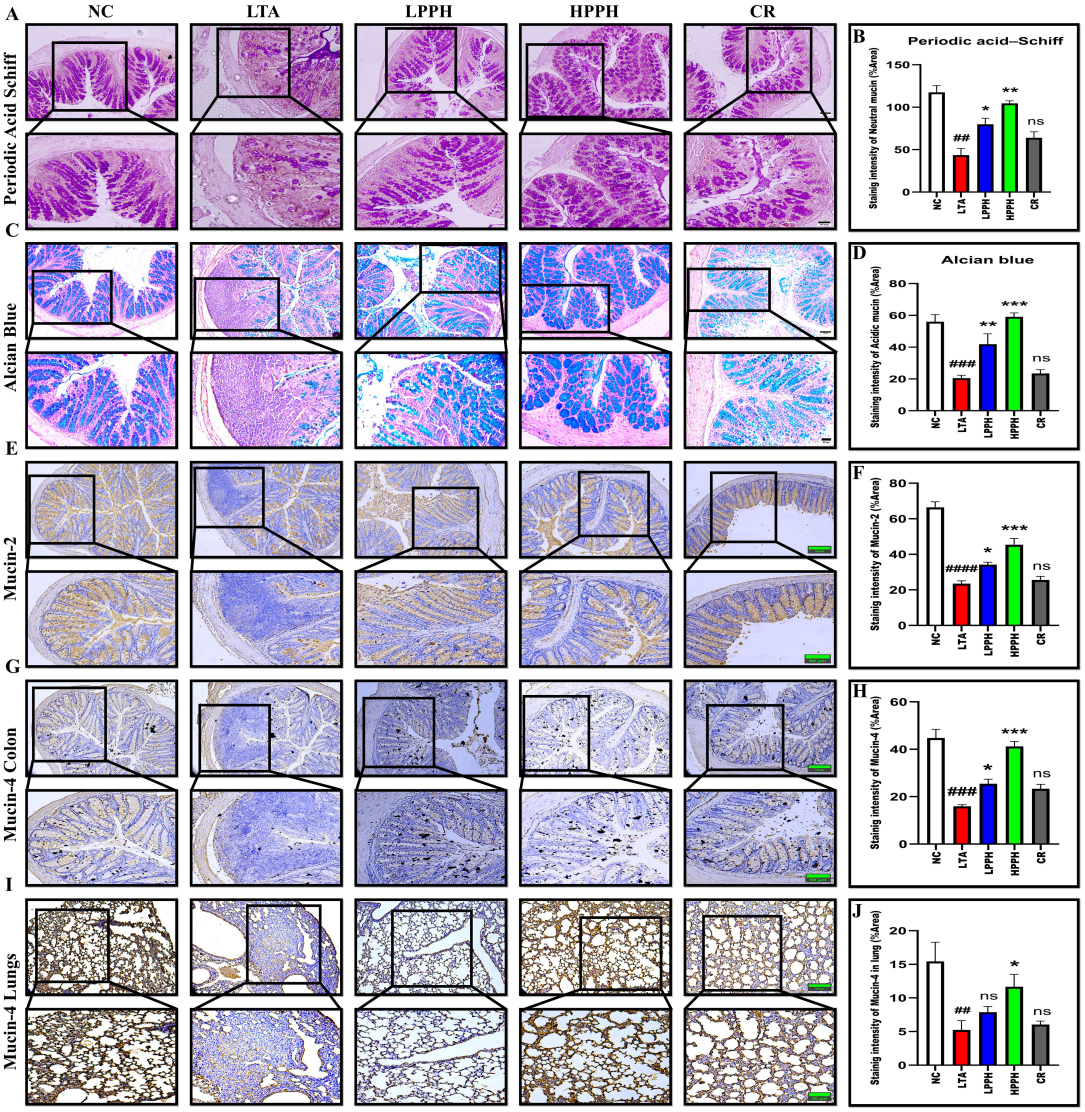
Histological analysis of mucin expression in colon and lung tissues using PAS, Alcian blue, and immunohistochemical staining. **(A)** PAS staining showing neutral mucins (magenta) in colon tissue across treatment groups; **(B)** Quantification of PAS-positive goblet cells; **(C)** Alcian blue staining showing acidic mucins (blue) in colon tissue; **(D)** Quantification of Alcian blue-positive areas; **(E)** Immunohistochemical staining for Mucin-2 expression in colon tissue; **(F)** Quantitative analysis of Mucin-2 staining intensity; **(G)** Immunohistochemical staining for Mucin-4 expression in colon tissue; **(H)** Quantitative analysis of colon Mucin-4 staining intensity; **(I)** Immunohistochemical staining for Mucin-4 expression in lung tissue; **(J)** Quantitative analysis of lung Mucin-4 staining intensity. Groups: NC (Normal Control), LTA (Lipoteichoic Acid), LPPH (Low-dose PPH), HPPH (High-dose PPH), CR (Crude extract). *p < 0.05, **p < 0.01, ***p < 0.001 vs. LTA group; ##*p* < 0.01, ###*p* < 0.001 vs. NC group, ####*p* < 0.0001; ns: not significant. Data presented as mean ± SEM (10 × and 20 × magnification; scale bars: 100 and 50 μm).

The colon’s epithelial barrier is protected by the intestinal mucus layer, which comprises acidic and neutral mucins produced by numerous goblet cells. To investigate the mucus layer’s thickness, goblet cell production, and mucin expression, Periodic Acid-Schiff (PAS) and Alcian blue (AB) staining were utilized. Analysis of PAS and AB staining revealed significant gut barrier damage in the LTA model, characterized by a thin mucus layer due to reduced goblet cell formation and decreased mucus production compared to the normal control group, as shown in Figures 4A,C. Conversely, PPH significantly enhanced goblet cell formation and mucin expression, resulting in improved mucus layer thickness in LPPH and HPPH groups. The PPH treatment groups exhibited results comparable to the normal control group, suggesting that PPH could promote mucus layer regeneration in LTA-treated mice. These results indicate that PPH may confer protection against the pneumonia induced by LTA in mouse models by increasing mucus thickness through enhanced goblet cell numbers and mucin production. Quantitative analysis of colon for PAS (Neutral mucin) and AB (Acidic mucin) staining shown in Figures 4B,D, where HPPH exhibited a more pronounced effect than LPPH>CR as compared to the model LTA group, respectively.

### 3.5 Effect of PPH on Mucin-2 and Mucin-4 in the Colon and Lungs

To evaluate the beneficial effects of the pupae peptide hydrolysate on mucin expression in LTA-treated mice, immunohistochemical staining was performed. The findings indicated the normal control group exhibited normal mucin expression, suggesting the presence of healthy functional goblet cells and an intact mucin layer in both the lungs and colon. Conversely, the group treated with LTA alone showed a marked reduction in Mucin-2 and Mucin-4 expression within these regions, indicating disrupted mucin secretion. This decrease in Mucin-2 and 4 expression aligns with known pathophysiological changes associated with pneumonia, including mucosal damage and inflammation. However, PPH treatment resulted in dose-dependent enhancement of Mucin-2 and 4 production. Between the PPH-supplemented groups, the HPPH>LPPH>CR group exhibited a substantial increase in Mucin-2 in the colon and Mucin-4 expression in both the lungs and colon, as shown in Figures 4E,G,I. Furthermore, Figures 4F,H present the quantitative examination of Mucin-2 and Mucin-4 in colonic tissue, while Figure 4J shows Mucin-4 in the lungs.

### 3.6 Immunofluorescent staining for tight junction protein

**Figure 5:**
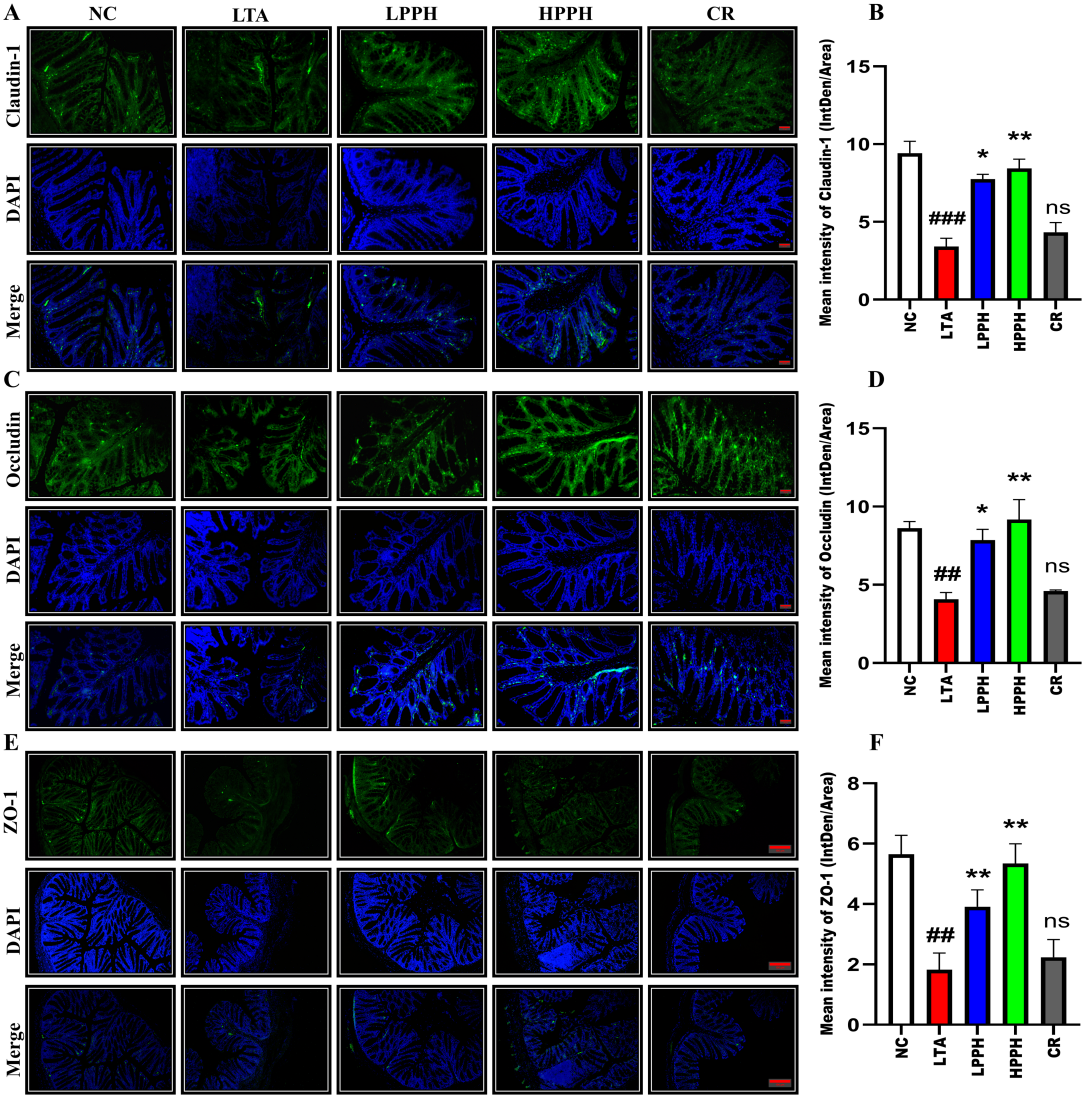
Representative immunofluorescence images showing the expression and distribution of tight junction proteins: **(A)** Claudin-1 (top row), **(C)** Occludin (middle row), and **(E)** ZO-1 (bottom row) across treatment groups: negative control (NC), lipoteichoic acid (LTA), low-dose PPH (LPPH), high-dose PPH (HPPH), and crude extract (CR). DAPI nuclear counterstaining is shown in blue, with merged images in the bottom panel of each set. Quantitative analysis of fluorescence intensity for **(B)** Claudin-1, **(D)** Occludin, and **(F)** ZO-1 expression. Data are presented as mean ± SEM; *p < 0.05, **p < 0.01 vs. LTA group, ##*p* < 0.01, ###*p* < 0.001compared to NC; ns: not significant. (10X with scale bar of 100 μm).

To examine the effects of LTA, the expression of Claudin-1 (CLDN-1), Occludin (OCC), and zonula occludens-1 (ZO-1) was assessed using immunofluorescent (IF) techniques. The model group exhibited a comparatively diminished expression as shown in Figures 5A,C,E suggesting that LTA had significantly disrupted the colon structures and reduced the number of cells responsible for CLDN-1, OCC, and ZO-1 secretion, in contrast to the control group. However, oral administration of varying doses of PPH (LPPH, HPPH, and CR) partially restored production, thereby improving colon architectural structures and enhancing the tight junction protein (TJS) expression compared to the model group. Notably, the high dose (HPPH) of PPH demonstrated the most significant improvement in TJS protein expression. Overall, these findings indicate the effectiveness of oral PPH treatment in alleviating LTA-induced pneumonia. Figures 5B,D,F show the quantification expression of CLDN-1, OCC, and ZO-1, respectively.

### 3.7 Regulation of serum cytokines by PPH

**Figure 6:**
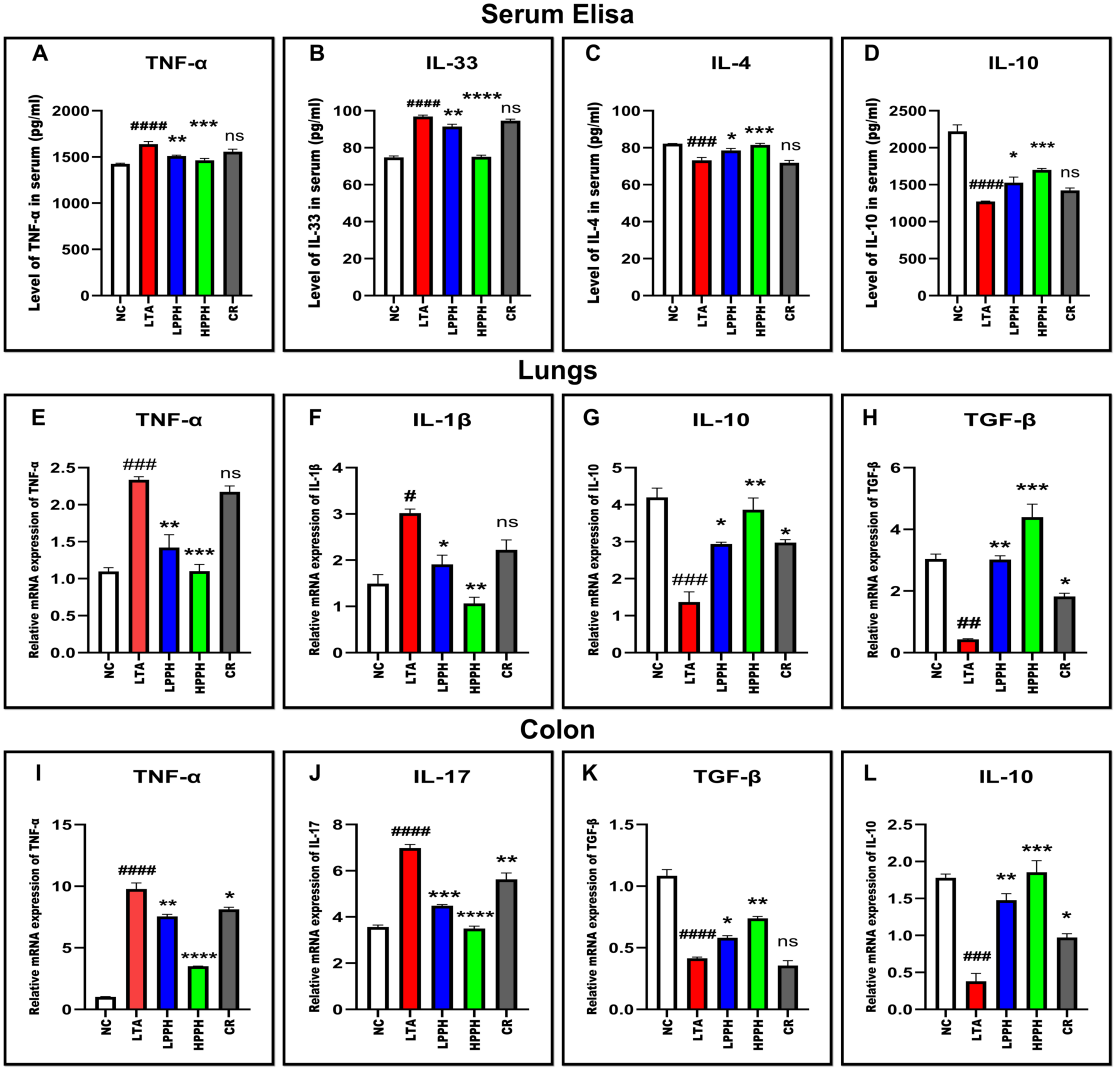
PPH treatment modulates pro- and anti-inflammatory cytokines in serum and in tissue samples. **(A–D)** Serum cytokine levels: TNF-α, IL-33, IL-4, and IL-10 respectively; **(E–H)** mRNA expression in lung tissue: TNF-α, IL-1*β*, IL-10 and TGF-β respectively; **(I–L)** mRNA expression in colon tissue: TNF-α, IL-17, TGF-β, and IL-10, respectively. Groups: NC (Normal Control), LTA (Lipoteichoic Acid), LPPH (Low-dose PPH), HPPH (High-dose PPH), CR (Crude extract). *p < 0.05, **p < 0.01, ***p < 0.001, ****p < 0.0001 vs. LTA group; #*p* < 0.05, ##*p* < 0.01, ###*p* < 0.001, ####*p* < 0.0001 vs. NC group; ns: not significant. Data presented as mean ± SEM.

To evaluate PPH therapeutic effects in an LTA-induced pneumonia mouse model, serum cytokine levels were quantified using ELISA. The results revealed significant changes in the concentrations of key inflammatory markers, providing valuable insights into immune response dynamics. The LTA group exhibited significantly elevated levels of pro-inflammatory cytokines, particularly TNF-*α* and IL-33 (*p* < 0.01), compared to the control. A 14-day PPH treatment significantly reduced TNF-α and IL-33 levels in the LPPH and HPPH groups (*p* < 0.05 and *p* < 0.01, respectively) as shown in Figures 6A,B, with HPPH demonstrating more pronounced effects. While the LTA group displayed decreased IL-10 and IL-4 levels, PPH treatment increased these anti-inflammatory cytokines (*p* < 0.01 and *p* < 0.001), notably in the HPPH group, as shown in Figures 6C,D. The CR treatment group exhibited some immunomodulatory effects for all the cytokines measured but did not reach statistical significance. These findings suggest that PPH modulates both pro-inflammatory and anti-inflammatory cytokine profiles in LTA-induced inflammation, with higher doses generally conferring greater therapeutic benefits.

### 3.8 RT-qPCR (quantitative real-time PCR) analysis

To elucidate the underlying molecular mechanisms of PPH’s therapeutic effects, we investigated the mRNA expression of inflammatory mediators in lung and colon tissues using RT-qPCR. In the lungs, the model LTA group exhibited significantly elevated levels of the pro-inflammatory cytokines TNF-*α* and IL-1β compared to the normal control group (*p* < 0.001 and *p* < 0.05, respectively). PPH treatment effectively attenuated this inflammatory response in a dose-dependent manner, with HPPH demonstrating a more potent suppression of these inflammatory markers (TNF-*α*, *p* < 0.001; IL-1β, *p* < 0.01) than LPPH (TNF-*α*, *p* < 0.01; IL-1β, *p* < 0.05) as shown in Figures 6E,F. In the CR treatment group, a slight modulation was noted, which was considered non-significant. The anti-inflammatory response in lung tissue was also evaluated based on TGF-β and IL-10 expressions. In the model LTA group with reduced levels of these protective cytokines, PPH treatment, particularly HPPH, significantly enhanced their expression (TGF-β, *p* < 0.001; IL-10, *p* < 0.01) compared to LPPH (TGF-β, p < 0.001; IL-10, *p* < 0.01) as shown in Figures 6G,H. The CR group also demonstrated a moderate increase in anti-inflammatory cytokine expression compared to the model LTA group in TGF-β and IL-10 (*p* < 0.05).

In the colon, a comparable pattern was observed, with the LTA group exhibiting markedly elevated expression of pro-inflammatory TNF-α and IL-17 (*p* < 0.0001). PPH treatment demonstrated significant immunomodulatory effects, with HPPH exhibiting the most substantial reduction in pro-inflammatory cytokine expression (TNF-*α* and IL-17, p < 0.0001), followed by LPPH (TNF-α, *p* < 0.01; IL-17, *p* < 0.001), including CR group (TNF-α, p < 0.05; IL-17, *p* < 0.01) as shown in Figures 6I,J. Notably, the expression of the anti-inflammatory mediators TGF-*β* and IL-10 was considerably augmented in the PPH-treated groups, with HPPH exhibiting superior effects (TGF-*β*, *p* < 0.01; IL-10, *p* < 0.001) compared to those of LPPH (TGF-β, *p* < 0.05; IL-10, *p* < 0.01) and CR (TGF-β, *p* > 0.05; IL-10, p < 0.05) as shown in Figures 6K,L. These findings suggest that PPH exerts its therapeutic effects by modulating both pro- and anti-inflammatory cytokine expression in lung and colon tissues, with higher doses demonstrating greater immunomodulatory potential.

### 3.9 PPH modulates inflammatory signaling pathways and TJS expression

**Figure 7:**
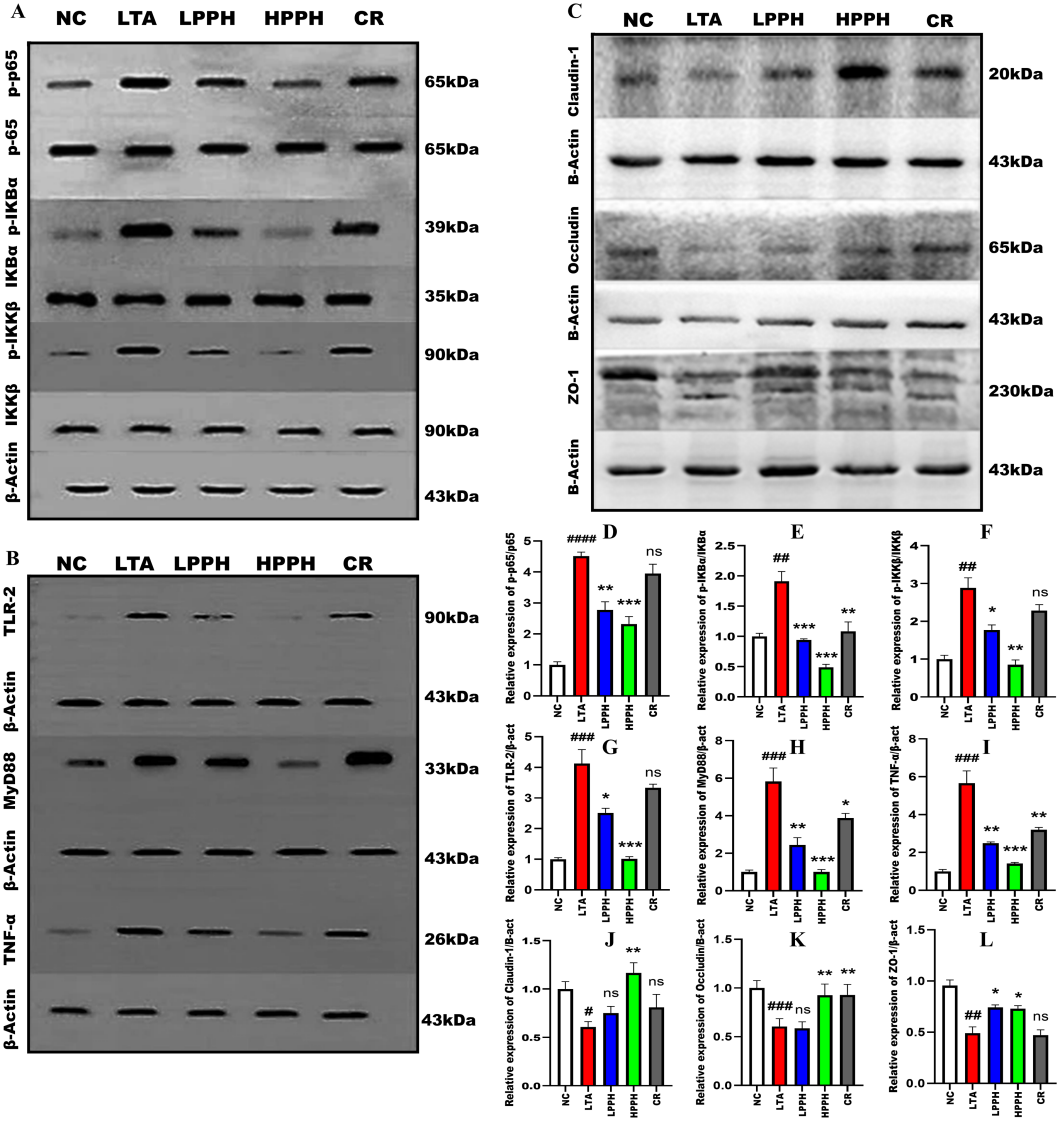
Effect of PPH on the modulation of signaling pathway and tight junction protein expression. **(A)** Analysis of NF-κB pathway proteins: p65, p-p65, IκB-α, p-IκB-α; **(B)** Analysis of TLR-2, MyD88, and TNF-α; **(C)** Analysis of tight junction proteins: Claudin-1, Occludin, and ZO-1 using β-actin as internal control; Quantitative analysis of **(D)** p-p65/p65; **(E)** p-IκB-α/IκB-α; **(F)**; p-IKKβ/IKKβ **(G)** TLR-2; **(H)** MyD88; **(I)** TNF-α; **(J)** Claudin-1; **(K)** Occludin; **(L)** ZO-1 relative protein expression levels. Groups: NC (Normal Control), LTA (Lipoteichoic Acid), LPPH (Low-dose PPH), HPPH (High-dose PPH), CR (Crude extract). Data acquired from three independent experiments and expressed as mean ± SEM. *p < 0.05, **p < 0.01, ***p < 0.001 vs. LTA group; #*p* < 0.05, ##*p* < 0.01, ###*p* < 0.001, ####*p* < 0.0001 vs. NC group.

To investigate the immunomodulatory effects of PPH on inflammatory signaling pathways, we examined several crucial factors. The LTA-treated group exhibited a significant increase in the phosphorylation of NF-κB p-65 (*p* < 0.0001), IKB-*α*, and IKK-β (*p* < 0.01), respectively, as shown in Figure 7, compared to the control group. However, PPH administration led to a substantial reduction in p-p65, particularly in HPPH (*p* < 0.001) and LPPH (*p* < 0.01), whereas the CR group was not significant, as shown in Figure 7 (A) and (D for quantification). Over 14 days, the phosphorylated IκB-α for both low and high doses of PPH had the same *p*-value (*p* < 0.001) while CR was (*p* < 0.01), as shown in Figure 7 (A) and (E for quantification). Similarly, the p-value for p-IKK-β high dose was (*p* < 0.01), low dose was (*p* < 0.05), while CR was not significant, as shown in Figure 7 (A) and (F for quantification), suggesting a downregulation of these signaling molecules. Furthermore, the TLR2 signaling pathway was also investigated; the LTA group exhibited altered protein expression levels of key components of the TLR2 pathway. The expressions of TLR2, MyD88, and TNF-α were significantly (*p* < 0.001) elevated in the LTA group compared with those in the normal group, respectively (Figure 7B). However, treatment with PPH significantly inhibits the TLR-2 pathway, particularly in the high-dose (*p* < 0.001), low-dose (*p* < 0.05, *p* < 0.01, *p* < 0.01), and CR groups (ns, *p* < 0.05, and *p* < 0.01), respectively, for TLR2, MyD88 and TNF-α as shown in Figure 7 (B) (quantitatively G, H and I). This suggested a considerable inhibitory effect.

Additionally, we evaluated the effects of PPH on tight junction proteins following LTA treatment. The LTA-treated group showed markedly decreased expression of CLDN-1, OCC, and ZO-1 compared to the normal group (*p* < 0.05, *p* < 0.001, 0.01). Notably, PPH treatment significantly enhanced the expression levels of CLDN-1, OCC, and ZO-1 especially in the high-dose group (*p* < 0.01, *p* < 0.01, *p* < 0.05), slightly in low-dose group (ns, ns, *p* < 0.05), and somehow in CR group (ns, *p* < 0.01, ns), respectively, as shown in Figure 7C (quantitatively J, K, and L), indicating a protective role of PPH on tight junction integrity.

### 3.10 PPH impact on intestinal microbial dynamics

**Figure 8:**
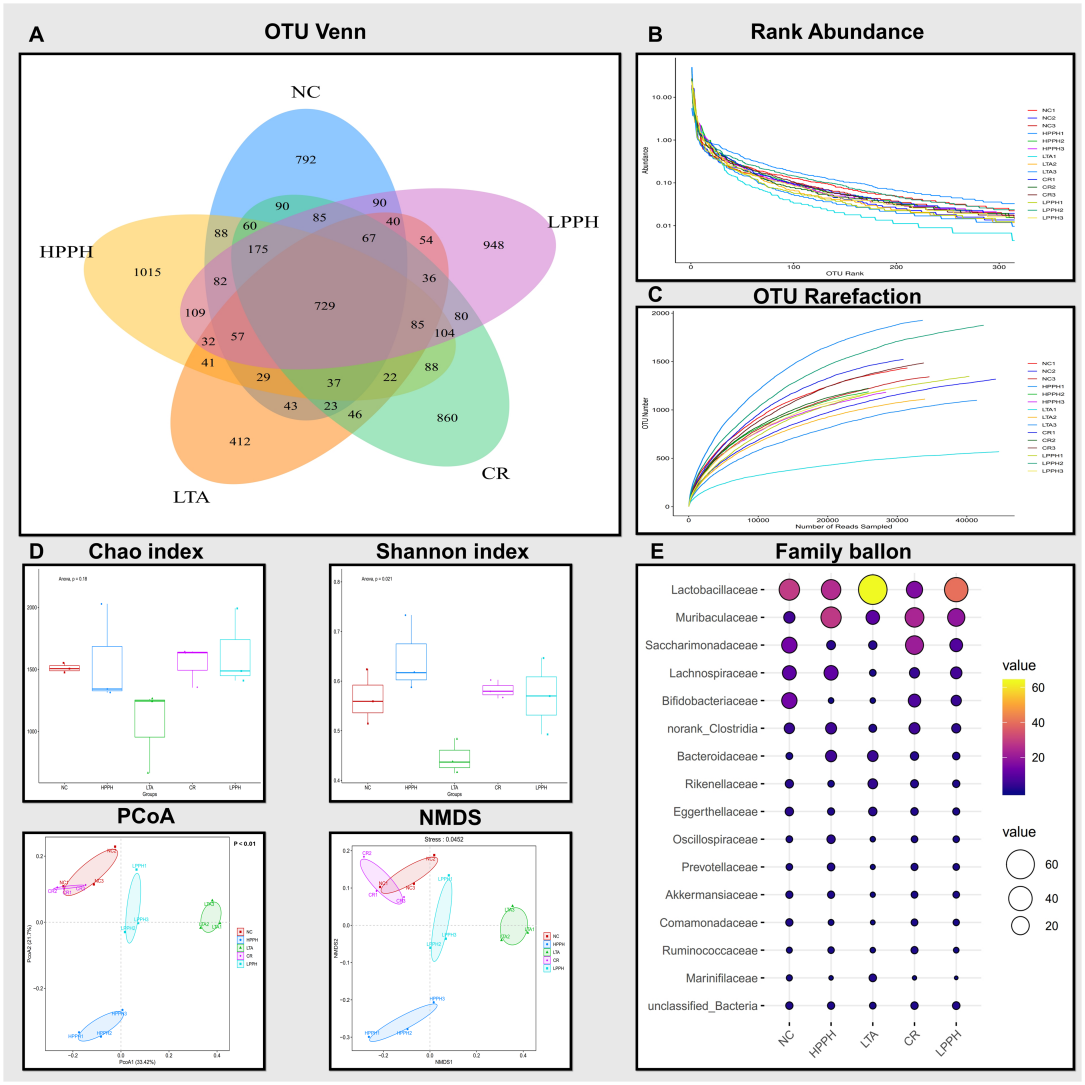
Analysis of gut microbiota composition and diversity across treatment groups. **(A)** Venn diagram showing shared and unique OTUs. **(B)** Rank abundance curves indicating species evenness. **(C)** OTU rarefaction curves showing sampling depth adequacy. **(D)** Alpha diversity indices: Chao and Shannon; Beta diversity indices: PCoA and NMDS plots showing sample clustering pattern. **(E)** Abundance of bacterial families across treatments groups. Groups: NC (Normal Control), LTA (Lipoteichoic Acid), LPPH (Low-dose PPH), HPPH (High-dose PPH), and CR (Crude extract).

PPH alters the microecology and abundance of gut microbiota at various levels. A comprehensive analysis of the bacterial diversity across groups was conducted. The Venn diagram illustrates the distribution and overlap of Operational Taxonomic Units (OTUs) across the NC, HPPH, LPPH, CR, and LTA groups. Unique OTUs were observed in the NC (792), LTA (412), LPPH (948), HPPH (1015), and CR (860) groups, as shown in Figure 8A. A core microbiome of 729 OTUs was shared among all the groups, indicating a stable core microbial community. The rank-abundance curve demonstrates the relative abundance distribution of the microbial species. The steep initial decline, followed by a long tail, indicates a few dominant and numerous rare species, suggesting a variable distribution of microbial abundance (Figure 8B). The OTU Rarefaction curves exhibited a relationship between sampling depth and observed OTU richness. The curves approached asymptotic plateaus at different levels, indicating an adequate sampling depth for most communities and varying species richness across samples, as depicted in Figure 8C. Furthermore, diversity indices and ordination, as shown by the Chao and Shannon indices, demonstrated alpha diversity variations, while PCoA and NMDS plots exhibited clustering patterns among groups (Figure 8D), suggesting distinct microbial community compositions. Additionally, the relative abundance of bacterial families was analyzed and visualized using balloon plots across the groups. Circle size and color intensity represent abundance levels, revealing distinct taxonomic profiles for each group, with some families exhibiting condition-specific enrichment or depletion patterns (Figure 8E). These results indicated that PPH significantly affected the microbial community composition, diversity, and structure, with shared and unique features across treatment groups.

**Figure 9:**
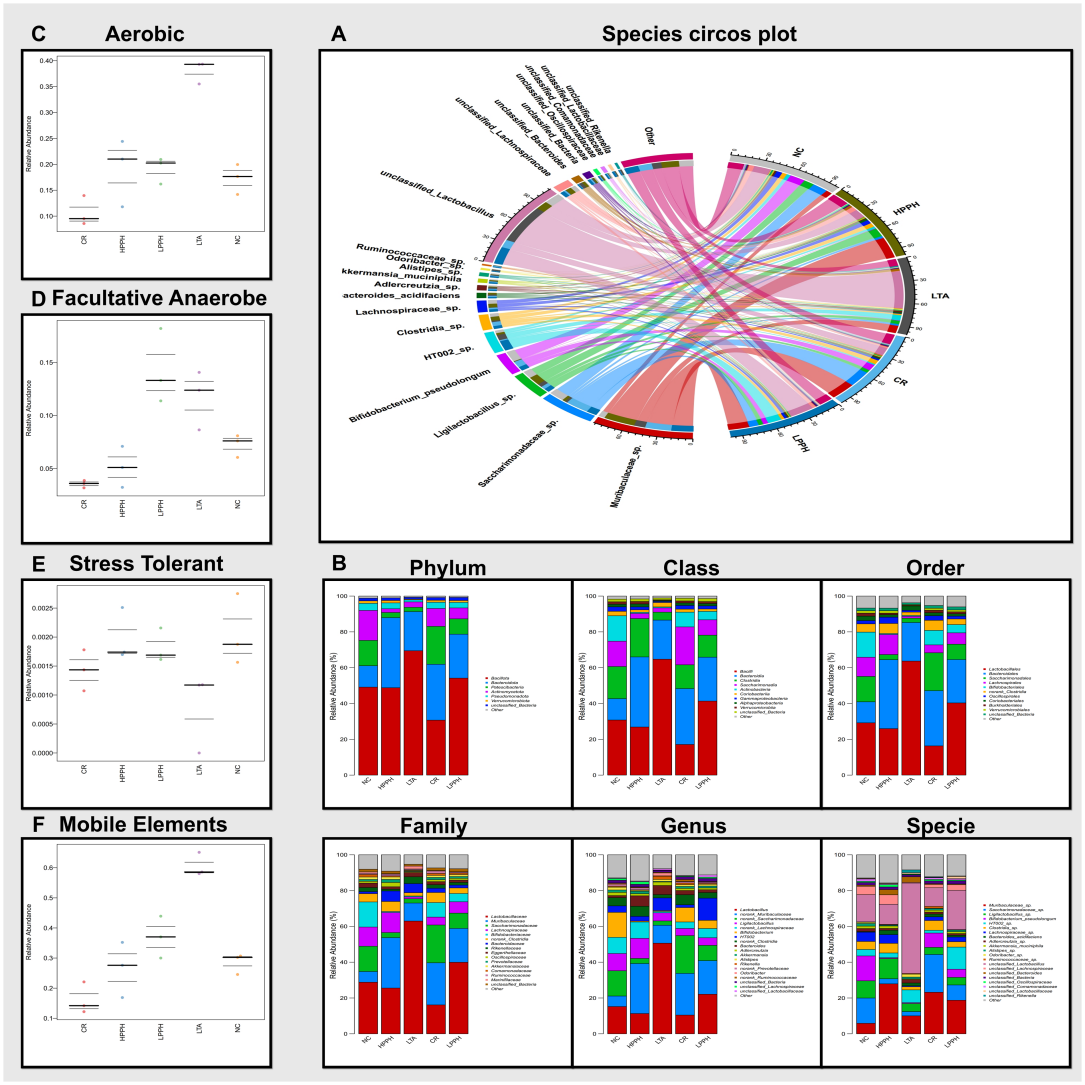
Analysis of gut microbiota composition and diversity across treatment groups. **(A)** Species circos plot; Colored bands represent the strength and direction of connections between taxonomic groups. **(B)** Relative abundance at different taxonomic levels (Phylum to Species). **(C–F)** Functional predictions showing relative abundance of aerobic, Facultative-anaerobes, stress-tolerant bacteria, and mobile genetic elements. Groups: NC (Normal Control), LTA (Lipoteichoic Acid), LPPH (Low-dose PPH), HPPH (High-dose PPH), and CR (Crude extract).

The chord diagram illustrates the distribution and interconnections of the bacterial species across different groups. This visual representation elucidates the associations between diverse bacterial species, including Lactobacillus, Saccharimonadaceae, Muribaculaceae, and Bifidobacteria. The width and color intensity of the connecting bands indicate the magnitude of the correlations between the bacterial taxa and their presence in the experimental groups, as shown in Figure 9A. The prominent bacterial species identified included *Bifidobacterium pseudolongum*, *Ligilactobacillus* sp., and multiple members of the *Ruminococcaceae*, suggesting their potential significance. This observation indicated that PPH treatment may influence the composition and abundance of specific bacterial populations. Furthermore, the bacterial diversity across taxonomic ranks revealed consistent microbiome changes in the experimental groups. Figure 9B illustrates the phylum-level distributions in the healthy control (NC), disease model (LTA), and PPH treatment (Low and High doses) groups. The most prevalent phyla in all groups were Bacillota, Bacteroidetes, Patescibacteria, Actinomycetota, Pseudomonadota, and Verrucomicrobiota with varying abundances. Bacillota (49.48%), Bacteroidetes (12.08%), Patescibacteria (14.06%), Actinomycetota (16.24%), Pseudomonadota (3.99%), and Verrucomicrobiota (1.42%) were predominant in the NC group. The LTA group exhibited an enormous increase in Bacillota (69.28%) and Bacteroidetes (21.89%), and decreases in Patescibacteria (2.39%), Actinomycetota (3.02%), and Pseudomonadota (1.23%), with negligible Verrucomicrobiota. In the LPPH group, Bacillota (53.84%) exhibited a slight decrease, whereas Bacteroidetes (25.07%), Patescibacteria (8.48%), Actinomycetota (6.05%), Pseudomonadota (3.04%), and Verrucomicrobiota (1.06%) increased compared to the LTA and approximated NC levels. HPPH displayed a reduction equivalent to that of NC in Bacillota (48.26%) and an increase in Bacteroidetes (39.92%) and Pseudomonadota (3.18%), whereas Patescibacteria (2.87%), Actinomycetota (2.02%), and Verrucomicrobiota (1.33%) remained comparable to those of LTA. The CR group exhibited a decrease in Bacillota (30.13%) and an increase in Bacteroidetes (31.44%), Patescibacteria (21.63%), Actinomycetota (9.88%), Pseudomonadota (3.47%), and Verrucomicrobiota (1.12%) relative to those in the LTA group. Bacterial class abundance analysis revealed significant variation (Figure 9B). The nine most prevalent classes in the bar plot were bacilli, bacteriidia, clostridia, Saccharimonadia, Actinobacteria, Coriobacteriia, Gammaproteobacteria, Alphaproteobacteria, and Verrucomicrobia, whereas the NC group exhibited a balanced microbiome, with bacilli (30.22%), Clostridia (18.81%), Saccharimonadia (14.04%), Actinobacteria (13.72%), and Bacteroidia (12.08%) being the most abundant, followed by smaller proportions of other classes and unclassified bacteria (1.49%). In contrast, the LTA group demonstrated dysbiosis, characterized by increases in Bacilli (64.47%) and Bacteroidia (21.89%) and decreased abundances of Clostridia (4.47%), Saccharimonadia (2.38%), and Gammaproteobacteria (0.66%), with Coriobacteriia (2.46%) remaining constant. Actinobacteria, Alphaproteobacteria, and Verrucomicrobia were absent from the LTA group. The LPPH group exhibited decreases in Bacilli (41.61%) and Coriobacteria (1.31%), and increased abundances of Bacteroidia (25.07%), Clostridia (11.69%), Saccharimonadia (8.47%), and Gammaproteobacteria (1.69%). Actinobacteria (4.69%), Alphaproteobacteria (1.33%), and Verrucomicrobia (1.06%) reappeared compared with the LTA group. The HPPH group demonstrated significant microbiome restoration, with bacilli (27.93%) and clostridia (19.81%) decreasing to the NC group levels, accompanied by other class changes. The CR group exhibited fluctuations, with Bacteroidia (31.44%), Saccharimonadia (21.62%), Bacilli (16.69%), Clostridia (13.13%), and Actinobacteria (8.18%) as the most prevalent genera. At the order level, Figure 9B, Lactobacillales exhibited a notably higher abundance in the LTA group (63.68%) than in the NC group (29.20%), with HPPH (25.95%), LPPH (40.31%), and CR (16.31%) demonstrating varying levels. Lachnospirales were the most abundant in the NC group (10.73%), followed by HPPH (11.22%), LPPH (6.35%), CR (4.41%), and LTA (1.06%). Family- and genus-level analyses (Figure 9B) confirmed significant differences between the NC- and LTA-treated groups, reflecting shifts in bacterial taxa abundance. Species-level analysis revealed increases in *unclassified_Lactobacillus* and *HT002_*sp., and reductions in *Bifidobacterium_pseudolongum*, *Clostridia* sp., *Lachnospiraceae sp*., *Akkermansia muciniphila*, and *unclassified_Lachnospiraceae*.

The analysis focused on aerobic, facultative anaerobic, stress-tolerant, and genetically mobile elements. The results demonstrated that the microbiomes in the normal control and PPH treatment groups exhibited relatively similar relative abundances across these phenotypes, suggesting that PPH treatment may substantially restore the functional profile of the microbiome, as shown in Figures 9C–F. In contrast, the LTA-treated group displayed notable variations in high-level phenotypes compared with the other groups. Specifically, the LTA-treated group exhibited a higher abundance of aerobic and genetic mobile elements, a decline in stress-tolerant bacteria, and variations in fecultative anaerobes. These findings indicate that LTA treatment may induce substantial shifts in the functional capabilities of the microbiome, potentially favoring the bacteria associated with these specific traits. The increased presence of mobile genetic elements could suggest enhanced horizontal gene transfer within the microbial community, whereas the higher abundance of aerobic bacteria might impact the interaction between the microbiome and the host.

LEfSe analysis identified distinct microbial signatures among the experimental groups (Supplementary Figure 2). In the HD group, there was a significant increase in *Bifidobacterium pseudolongum*, *Bifidobacterium*, and *Lactobacillus* spp., taxa recognized for their role in enhancing gut barrier function and mitigating inflammatory responses. The LD treatment facilitated the proliferation of Odoribacter and members of *Marinifilaceae*, whereas the NC group exhibited a high prevalence of *Muribaculaceae* spp., indicative of a stable gut microbiota. In contrast, the PC group demonstrated elevated levels of *Clostridia_UCG_014*, associated with pro-inflammatory gut conditions, and CR treatment increased *Candidatus Saccharimonas*, a taxon frequently linked to opportunistic growth. These patterns, corroborated by LDA scores exceeding 4.0, suggest that PPH, particularly at higher doses, contributes to the restoration of microbial balance by enhancing beneficial commensals and reducing dysbiotic taxa associated with pneumonia.

To examine the variations in taxa between the low-dose and high-dose groups, we analyzed genus-level abundance, with the results illustrated in a heatmap (Supplementary Figure 3). This figure presents the relative abundance patterns of key genera across all treatment groups, including NC, PC (LTA), LPPH (low-dose PPH), HPPH (high-dose PPH), and CR. Importantly, the high-dose PPH treatment was correlated with an increased presence of *Ligilactobacillus*, *norank_Lachnospiraceae*, *norank_Muribaculaceae*, *norank_Clostridia*, *Bacteroides*, *Akkermansia*, *norank_Prevotellaceae*, *unclassified_Lachnospiraceae*, as well as a decreased presence of *norank_Saccharimonadaceae*, *Bifidobacterium*, *HT002*, *unclassified_Bacteria*, *unclassified_Lactobacillaceae*, *Bacteroides*, and *Alistipes* when compared to the low-dose group, indicating a dose-dependent effect on the gut microbiota. Comprehensive gut microbiome analysis across taxonomic levels suggests that the disease impacts microbiome composition. The LTA group likely represents dysbiosis compared with the NC group. Both LPPH and HPPH treatments demonstrated a trend towards reversing this shift, with high-dose treatment exhibiting a more pronounced effect. This indicates that HPPH was more effective in modulating gut microbiota, restoring the balance closer to normal control levels, and alleviating dysbiosis.

## 4 Discussion

Lower respiratory infections, especially pneumonia, significantly impact global morbidity and mortality rates (46). Severity is pronounced in developing regions with limited treatment options and is exacerbated by inadequate healthcare in wealthy nations. Worldwide, 50% of the population lacks essential health services, and healthcare costs drive 100 million people into poverty annually (47). Bacterial infections have contributed to these problems. Our research identified *Enterococcus faecalis*, isolated from the intestines of children with pneumonia, as a trigger for inflammatory immune responses in the lung tissue, with lipoteichoic acid (LTA) being the primary virulence factor, highlighting the gut-lung axis (1). Research has demonstrated the potential of natural compounds in treating inflammatory conditions, highlighting the efficacy of polysaccharides derived from mushrooms, plant extracts, and peptides (7, 48, 49). Public interest in safe and effective products aligns with research on food-derived bioactive peptides, which known to reduce blood pressure, prevent coagulation, regulate immune functions, and counteract oxidative stress (50, 51).

Our study examined the immunomodulatory properties of enzymatically produced *Vespa orientalis* pupae peptide hydrolysate (PPH) using bromelain. Analysis on LC–MS revealed a wide range of peptides with distinct molecular weights and amino acid sequences, consistent with previous research on shrimp hydrolysates analyzed using MALDI-TOF (52).

Insect-derived bioactive components are promising alternatives to traditional peptide sources. Although venom from *Vespa* species has shown anticancer potential, its non-selective nature raises concerns for healthy cells (29, 53). In contrast, the larval and pupal stages demonstrate anti-inflammatory, immunomodulatory, and gut microbiota-restoring properties with reduced toxicity. Antioxidant and cytotoxic effects have been observed in aqueous pupal extracts of *V. affinis* and larval hemolymph of *M. domestica* (10, 26). This study provides the first evidence that *V. orientalis* pupae peptide hydrolysate (PPH) exhibits anti-inflammatory and immunomodulatory effects in an LTA-induced pneumonia mouse model, gut microbiota restoration, indicating its therapeutic potential.

Our analysis highlighted the high protein content in PPH and the variety of enzymatically generated bioactive peptides. The amino acid profile is essential for the biological functions of these peptides. For example, lysine enhances growth, fortifies the immune system, and has antiviral properties (54). Lysine deficiency can impair cytokine production and immune responses (55). Additionally, aspartic acid, glutamic acid, and serine are crucial for energy production, cognitive function, and immune maintenance (56–58), including gut microbiota-derived butyric acid which regulates calcific aortic valve disease pathogenesis (59).

In our LTA-induced pneumonia model, PPH treatment significantly improved clinical symptoms. Disease Activity Index (DAI) and Pneumonia Severity Index evaluations showed notable progress, especially with higher PPH doses. The gut-lung axis was notably affected, with PPH-treated mice exhibiting reduced lung inflammation and increased colon length. These findings highlight the anti-inflammatory and immunomodulatory properties of PPH, suggesting it as a promising therapeutic option.

The digestive and respiratory systems form an intricate and ever-changing ecosystem wherein the host organism and its microbial population interact in a subtle equilibrium to preserve homeostasis. The host’s protective mechanisms, encompassing both innate and adaptive immune responses, are crucial in identifying and neutralizing potential threats, such as pathogenic microbes. An imbalance in this fragile system can trigger the onset of inflammatory conditions (60), subsequently resulting in lung inflammation (1). This interlinked network underscores the importance of investigating both the localized and systemic impacts of inflammation, as further explored in the context of LTA-induced damage.

The administration of LTA resulted in notable structural damage, inflammation, and epithelial damage to the colon and lung, corroborating the research of Zadeh, Khan et al. (61) on their model of inflammatory intestinal disease. Conversely, PPH treatment exhibited therapeutic advantages, with higher dosages yielding superior colon structural regeneration and a significant reduction in inflammation, including enhanced mucosal repair. These results are consistent with those reported by Wang et al. (62), who observed similar protective effects of polyphenols on intestinal barrier function. In the pulmonary system, LTA induces substantial inflammatory changes, including heightened cellular infiltration and modified alveolar structures (1). PPH treatment significantly mitigated these pathological alterations, particularly at elevated doses. The dose-dependent efficacy of PPH treatment implies a shared mechanism involving anti-inflammatory pathways, which also encompasses the physical and functional defenses of the colon. Moreover, the lung and colon mechanical element serves as a crucial physical and functional defense mechanism. This system comprises mucus layers and epithelial cells that are tightly bound by junction proteins. The outer mucus layer, consisting primarily of water (>98%) and mucins secreted by goblet cells, plays a vital role in protecting the underlying intestinal epithelial cells (63, 64). Our findings suggest that LTA affects both the colon and lungs by damaging the mucosal structure, lowering tight junction protein expression, and reducing the number of goblet cells that produce mucin. However, PPH supplementation exhibited dose-dependent therapeutic effects, significantly alleviating these pathological changes and restoring the normal and healthy structures in the treated groups. These benefits are also apparent in the regeneration of tight junction proteins.

The integrity of the epithelial barrier relies on tight junction proteins that form cell adhesion complexes, separating the apical and basolateral membrane domains. Immunofluorescence analysis revealed significant changes in the expression and distribution of key tight junction proteins Claudin-1, Occludin, and ZO-1, which are crucial for selective molecular diffusion and serve as predictive indicators (64). The proper configuration and maintenance of these proteins are essential for intestinal balance and pathogen defense. LTA-induced damage to tight junction proteins was evident by reduced immunoreactivity, discontinuous membrane staining, and lower fluorescence intensity, indicating a compromised barrier function. This decline in protein expression due to LTA was associated with increased intestinal permeability and inflammation, highlighting the importance of these proteins in maintaining mucosal barrier function. PPH supplementation showed significant protective effects by restoring tight junction protein expression. Enhanced expression and proper membrane localization of Claudin-1, Occludin, and ZO-1 with PPH treatment suggest stabilization of the epithelial barrier complex. These findings concur with those of (65), who reported similar restoration by *Scorias spongiosa* polysaccharides. The protective mechanism likely involves the regulation of inflammatory pathways and the enhancement of tight junction protein expression. These results strongly suggest that PPH supplementation, especially at higher doses, effectively counteracts LTA-induced pathological changes and restores colon barrier integrity by modulating tight junction protein expression, thus representing a therapeutic target for intestinal barrier function under inflammatory conditions. PPH to restore tight junction protein expression complements its regulation of cytokine-mediated immune responses.

Cytokines play various roles in the regulation of immune responses and disease progression during lung inflammation. Pro-inflammatory cytokines, such as TNF-*α*, IL-1*β*, IL-33, and IL-17, primarily drive lung inflammation by triggering immune responses, attracting inflammatory cells, and releasing inflammatory mediators (65, 66). Conversely, anti-inflammatory cytokines, such as TGF-β, IL-4, and IL-10, counteract pro-inflammatory responses by reducing excessive inflammation and promoting tissue repair and homeostasis; they are crucial for regulating mucus metabolism and lung function (67). Di Tommaso et al. (64) highlight the importance of the balance between pro- and anti-inflammatory cytokines in determining lung inflammation’s course and severity. Understanding this balance is vital for the development of therapies for inflammatory lung disease. Our study on LTA-induced pneumonia showed a significant imbalance in cytokine expression, with elevated levels of pro-inflammatory cytokines (TNF-α, IL-1β, IL-33, and IL-17) and reduced levels of anti-inflammatory markers (IL-10, IL-4, and TGF-β), creating a persistent inflammatory environment. PPH has emerged as a promising therapeutic, demonstrating a dose-dependent effect as higher PPH doses progressively reduce pro-inflammatory cytokines and increase anti-inflammatory cytokines. This correlation is closely aligned with (37, 68), which indicated that peptides and polysaccharides have immunomodulatory capabilities and potential to rebalance the immune response.

Nuclear factor kappa B (NF-κB) proteins play a crucial role in regulating various physiological processes, including immune responses, apoptosis, cell proliferation, inflammation, and oncogenesis (69–71). Aberrations in NF-κB activity, whether caused by genetic alterations or disruptions in its transcriptional function, have been associated with the onset of leukemia, lymphomas, and solid tumors (72, 73). This highlights the significance of comprehending NF-κB signaling pathways in disease progression and potential therapeutic strategies. Given the therapeutic promise of PPH in mitigating LTA-induced pneumonia in mice, we explored the inflammation-related signaling pathways linked to pneumonia. Earlier research has established the vital roles of the TLR2 and NF-κB signaling pathways in pneumonia progression (74, 75). Our research demonstrated that PPH achieves its therapeutic effects in pneumonia through the dephosphorylation of NF-κB, TLR2, and MyD88 in the treated groups. This implies that PPH may modulate these essential signaling molecules, potentially dampening the inflammatory response and reducing the severity of pneumonia, which is corroborated in the gut-lung axis and microbiome.

The gut microbiome is a vital ecosystem that supports overall health across various systems. Dysbiosis, an imbalance in gut microbes, is linked to health issues such as neuropsychiatric disorders, diabetes, and cancer including difficulties in diagnosis (76–80). The gut hosts over 100 trillion bacteria and three million unique genes (81), primarily Firmicutes, Bacteroidetes, Proteobacteria, and Actinobacteria (82). Gut microbiotas regulate the immune response, intestinal balance, and overall well-being through interactions with host immunity (83). The lung microbiome is smaller and more variable and is influenced by microbial migration and host defenses (84). Healthy lungs maintain microbial balance through micro-respiration from the upper respiratory tract (85). Pulmonary health issues can disrupt the lung microbiome and increase microbial populations, sometimes in the gastrointestinal system (86). Our research supports (82), identifying Firmicutes, Bacteroidetes, and Actinomycetota as the dominant phyla. This study examined the prebiotic effects of PPH on LTA-induced dysbiosis. Dysbiosis, marked by microbial community imbalance, can cause health problems (87). The LTA group showed significant changes in bacterial diversity, indicating dysbiosis. The model group formed distinct clusters from the control group, showing an altered microbial composition due to LTA. PPH treatment, especially at higher doses, reversed dysbiosis by increasing bacterial species richness. The microbial profile of the PPH-treated group resembled that of the control group, suggesting a prebiotic effect, aligning with (37, 88). These findings indicate that PPH may restore and maintain a healthy gut microbiome, offering therapeutic potential for dysbiosis-related conditions. Our findings indicated regulation at the family level within *Lactobacillaceae* of the model LTA group, exceeding 1–5% occurrence, which is characteristic of small intestinal bacterial overgrowth (SIBO). This overgrowth of facultative anaerobes, which produce enterotoxins and cause epithelial damage, has been observed (89, 90). However, this was reduced in the PPH-treated groups. The elevated levels of *Muribaculaceae* in the PPH-treated group were associated with the production of short-chain fatty acids (SCFAs), such as acetate, propionate, and butyrate, which are essential for intestinal health (91, 92). *Saccharimonadaceae*, known as epibionts that influence other gut microorganisms (93), were also replenished with low-dose PPH. Similarly, in *Lachnospiraceae*, fermenting fibers into SCFAs, particularly anti-inflammatory butyrate (92), was restored at high doses of PPH. Lastly, the replenishment of Bifidobacteriaceae, which is capable of fermenting carbohydrates, oligosaccharides, and SCFAs, contributes to anti-inflammatory and anti-allergic responses (94, 95). Furthermore, analysis of phenotypic differences among the experimental groups provides insights into the functional characteristics of the microbiome. The analysis focused on several key phenotypes, including aerobic, facultative anaerobic bacteria, the presence of mobile genetic elements, and stress-tolerant bacteria, which are a key component of health status (96). The results demonstrated that the microbiome phenotypes in the normal control and PPH treatment groups exhibited relatively similar relative abundances across these phenotypes, suggesting that PPH treatment may substantially restore the functional profile of the microbiome which is closely aligned to (52, 68). In contrast, the disease model group displayed notable variations in high-level phenotypes compared with the other groups. Specifically, a higher abundance of functional genes associated with mobile genetic elements, facultative anaerobes, and aerobic bacteria. These findings indicate that LTA treatment may induce substantial shifts in the functional capabilities of the microbiome, potentially favoring bacteria associated with these specific traits. The increased presence of mobile genetic elements could suggest enhanced horizontal gene transfer within the microbial community, while the higher abundance of aerobic bacteria might impact the interaction of the microbiome with the host. The shift in bacteria underscores the significant effect of PPH treatment on the composition and functional profile of the microbiome.

The findings of this study on *Vespa orientalis* pupae peptide hydrolysate (PPH) revealed its multifaceted potential in combating LTA-induced pneumonia. By facilitating the restoration of intestinal epithelial integrity, PPH may help to maintain the crucial barrier function of the gut, preventing the translocation of harmful bacteria and toxins. This restoration process is likely complemented by PPH ability to promote a favorable shift in gut microbiota composition, potentially enhancing the population of beneficial bacteria that contribute to overall immune health and resilience against respiratory infections. Furthermore, the capacity of PPH to modulate immune responses and attenuate systemic inflammation suggests its broader impact on the body’s defense mechanisms. This dual action could be particularly beneficial in the context of pneumonia, in which an overactive immune response often exacerbates tissue damage. By fine-tuning the immune system and reducing excessive inflammation, PPH may help strike a balance between fighting off the infection and minimizing collateral damage to the lung tissue. However, a limitation of the present study is that PPH was used as a crude hydrolysate containing multiple peptides and other components, which prevents precise identification of the specific bioactive peptides responsible for the observed effects. Future studies should focus on fractionating, isolating, and characterizing individual peptides, incorporating appropriate control groups, and conducting in-depth mechanistic investigations to clarify their specific contributions and molecular pathways. These combined effects position PPH as a promising therapeutic candidate that addresses multiple aspects of LTA-induced pneumonia pathogenesis, potentially offering a more comprehensive treatment approach than conventional therapies that may target only one or two of these mechanisms.

## 5 Conclusion

In conclusion, this study demonstrates the significant therapeutic potential of peptide hydrolysates derived from *Vespa orientalis* pupae in the treatment of LTA-induced pneumonia. The multifaceted effects of PPH indicate its potential as a comprehensive therapeutic intervention, targeting not only pulmonary inflammation but also the associated aspects of gut health and immune system function. PPH offers a holistic treatment approach by attenuating inflammation, enhancing intestinal barrier integrity, modulating immune responses, restoring gut microbiome homeostasis, and acknowledging the intricate connection between respiratory wellness and gastrointestinal function. These findings have significant implications for clinical practice and future research. This investigation underscores the importance of considering the gut-lung axis in managing respiratory illnesses, suggesting that therapies targeting intestinal health could be crucial in treating pneumonia and other inflammatory lung disorders. This perspective represents a paradigm shift in pneumonia treatment, extending beyond conventional antibiotic therapies to encompass a more comprehensive understanding of systemic health. This field of study may lead to the development of innovative therapeutic strategies that leverage the interconnectedness of various physiological systems to achieve more efficacious and comprehensive treatment outcomes for respiratory diseases.

## Data Availability

The original contributions presented in the study are included in the article/Supplementary material; further inquiries can be directed to the corresponding author.

## References

[1] Tian Z Khan AI Rehman AU Deng T Ma C Wang L. Virulence factors and mechanisms of paediatric pneumonia caused by *Enterococcus faecalis*. Gut Pathog. (2023) 15:2. doi: 10.1186/s13099-022-00522-z, PMID: 36624474 PMC9830894

[2] Abu Gazia M El-Magd MA. Ameliorative effect of cardamom aqueous extract on doxorubicin-induced cardiotoxicity in rats. Cells Tissues Organs. (2019) 206:62–72. doi: 10.1159/000496109, PMID: 30716735

[3] Kozłowski J Kozłowska A Kocki J. Breast cancer metastasis-insight into selected molecular mechanisms of the phenomenon. Postepy Hig Med Dosw. (2015) 69:447–51. doi: 10.5604/17322693.1148710, PMID: 25897105

[4] El-Magd MA Mohamed Y El-Shetry ES Elsayed SA Gazia MA Abdel-Aleem GA. Melatonin maximizes the therapeutic potential of non-preconditioned MSCs in a DEN-induced rat model of HCC. Biomed Pharmacother. (2019) 114:108732. doi: 10.1016/j.biopha.2019.108732, PMID: 30925457

[5] Awad MG Ali RA Abd El-Monem DD El-Magd MA. Graviola leaves extract enhances the anticancer effect of cisplatin on various cancer cell lines. Mol Cell Toxicol. (2020) 16:385–99. doi: 10.1007/s13273-020-00092-8

[6] Attia AM Khodair AI Gendy EA El-Magd MA Elshaier YAMM. New 2-oxopyridine/2-thiopyridine derivatives tethered to a benzotriazole with cytotoxicity on MCF7 cell lines and with antiviral activities. Lett Drug Des Discov. (2020) 17:124–37. doi: 10.2174/1570180816666190220123547

[7] Bin Y Peng R Lee Y Lee Z Liu Y. Efficacy of Xuebijing injection on pulmonary ventilation improvement in acute pancreatitis: a systematic review and meta-analysis. Front Pharmacol. (2025) 16:1549419. doi: 10.3389/fphar.2025.1549419, PMID: 40308770 PMC12041077

[8] Luo J Li X Zhang L Deng M Zhao J Zhang J. 5-deoxy-rutaecarpine protects against LPS-induced acute lung injury via inhibiting NLRP3 inflammasome-related inflammation. Front Pharmacol. (2025) 16:1522146. doi: 10.3389/fphar.2025.1522146, PMID: 39981175 PMC11841402

[9] Pettit GR Meng Y Herald DL Knight JC Day JF Agents A. 553. The Texas grasshopper Brachystola m agna. J Nat Prod. (2005) 68:1256–8. doi: 10.1021/np0402367, PMID: 16124772 PMC3251507

[10] El-Garawani I El-Seedi H Khalifa S El Azab IH Abouhendia M Mahmoud S. Enhanced antioxidant and cytotoxic potentials of lipopolysaccharides-injected *musca domestica* larvae. Pharmaceutics. (2020) 12:1111. doi: 10.3390/pharmaceutics12111111, PMID: 33227988 PMC7699146

[11] Zhou Y Li L Yu Z Gu X Pan R Li Q. *Dermatophagoides pteronyssinus* allergen Der p 22: cloning, expression, IgE-binding in asthmatic children, and immunogenicity. Pediatr Allergy Immunol. (2022) 33:e13835. doi: 10.1111/pai.13835, PMID: 36003049

[12] Yan Y-M Li L-J Qin X-C Lu Q Tu Z-C Cheng Y-X. Compounds from the insect Blaps japanensis with COX-1 and COX-2 inhibitory activities. Bioorg Med Chem Lett. (2015) 25:2469–72. doi: 10.1016/j.bmcl.2015.04.085, PMID: 25980909

[13] An C Li D Du R. Analysis of antibacterial-relative proteins and peptides in housefly larvae. J Hyg Res. (2004) 33:86–8. 15098487

[14] Guo G Tao R Li Y Ma H Xiu J Fu P. Identification and characterization of a novel antimicrobial protein from the housefly *Musca domestica*. Biochem Biophys Res Commun. (2017) 490:746–52. doi: 10.1016/j.bbrc.2017.06.112, PMID: 28645609

[15] Di Mattia C Battista N Sacchetti G Serafini M. Antioxidant activities in vitro of water and liposoluble extracts obtained by different species of edible insects and invertebrates. Front Nutr. (2019) 6:106. doi: 10.3389/fnut.2019.00106, PMID: 31380385 PMC6643021

[16] Zielińska E Karaś M Jakubczyk A. Antioxidant activity of predigested protein obtained from a range of farmed edible insects. Int J Food Sci Technol. (2017) 52:306–12. doi: 10.1111/ijfs.13282

[17] Huis A. Itterbeeck J. V. Klunder H. Mertens E. Halloran A. Muir G.. Edible insects: future prospects for food and feed security. Rome, Italy: Food and Agriculture Organization of the United Nations (FAO) (2013).

[18] Ma Q Qian Y Su W Shi L Wang E Yu A. Degradation of agricultural polyethylene film by greater wax moth (galleria mellonella) larvae and screening of involved gut bacteria. Ecotoxicol Environ Saf. (2025) 303:118841. doi: 10.1016/j.ecoenv.2025.118841, PMID: 40829281

[19] Abu Khudir RH El-Ghannam MA Salama AF Tousson EM El-Dsoki SM. Curcumin attenuated oxidative stress and inflammation on hepatitis induced of by fluvastatin in female albino rats. Alexandria J Vet Sci. (2019) 62. doi: 10.5455/ajvs.48116

[20] Haddad NJ Fuchs S Haddaden J Kopelke J-P. Record of *Sphecophaga vesparum* Curtis, a natural enemy of *Vespa orientalis* in northern Jordan. Zool Middle East. (2005) 35:114–6. doi: 10.1080/09397140.2005.10638117

[21] Bagriacik N. Determination of some structural features of the nest paper of *Vespa orientalis* Linneaus, 1771 and *Vespa crabro* Linneaus, 1758 (Hymenoptera: Vespinae) in Turkey. Arch Biol Sci. (2011) 63:449–55. doi: 10.2298/ABS1102449B

[22] Dehghani R Kassiri H Mazaheri-Tehrani A Hesam M Valazadi N Mohammadzadeh M. A study on habitats and behavioral characteristics of hornet wasp (Hymenoptera: Vespidae: *Vespa orientalis*), an important medical-health pest. Biomed Res. (2019) 30:61–6. doi: 10.35841/biomedicalresearch.30-18-1187

[23] Ebrahimi E Carpenter JM. Distribution pattern of the hornets *Vespa orientalis* and *V. crabro* in Iran: (Hymenoptera: Vespidae). Zool Middle East. (2012) 56:63–6. doi: 10.1080/09397140.2012.10648942

[24] Ishak HD Miller JL Sen R Dowd SE Meyer E Mueller UG. Microbiomes of ant castes implicate new microbial roles in the fungus-growing ant *Trachymyrmex septentrionalis*. Sci Rep. (2011) 1:204. doi: 10.1038/srep00204, PMID: 22355719 PMC3244503

[25] Narzari S Sarmah J. Proximate composition of wild edible insects consumed by the Bodo tribe of Assam, India. Int J Bioassays. (2015) 4:4050–4. doi: 10.21746/IJBIO.2015.07.002

[26] Dutta P Dey T Manna P Kalita J. Antioxidant potential of Vespa affinis L., a traditional edible insect species of north East India. PLoS One. (2016) 11:e0156107. doi: 10.1371/journal.pone.0156107, PMID: 27195807 PMC4873131

[27] Jalaei J Fazeli M Rajaian H Shekarforoush SS. In vitroantibacterial effect of wasp (Vespa orientalis) venom. J Venomous Anim Toxins Trop Dis. (2014) 20:22–06. doi: 10.1186/1678-9199-20-22, PMID: 24955088 PMC4045935

[28] Lee SH Baek JH Yoon KA. Differential properties of venom peptides and proteins in solitary vs. social hunting wasps. Toxins. (2016) 8:32. doi: 10.3390/toxins8020032 26805885 PMC4773785

[29] Mukund H Manjunath P. Comparative enzyme activity of *Vespa orientalis* venom and its photooxidized venom products. J Biochem Biophys. (2017) 1:104. doi: 10.15744/2576-7623.1.104

[30] Moreno M Zurita E Giralt E. Delivering wasp venom for cancer therapy. J Control Release. (2014) 182:13–21. doi: 10.1016/j.jconrel.2014.03.005, PMID: 24631864

[31] Galante P Campos GA Moser JC Martins DB dos Santos Cabrera MP Rangel M. Exploring the therapeutic potential of an antinociceptive and anti-inflammatory peptide from wasp venom. Sci Rep. (2023) 13:12491. doi: 10.1038/s41598-023-38828-w 37528129 PMC10393941

[32] Gao Y Yu W-X Duan X-M Ni L-L Liu H Zhao H-R. Wasp venom possesses potential therapeutic effect in experimental models of rheumatoid arthritis. Evid Based Complement Alternat Med. (2020) 2020:6394625. doi: 10.1155/2020/6394625, PMID: 32328136 PMC7165351

[33] Saidemberg D. M., daL. C.Silva-Filho, Tognoli L. M. C. Tormena C. F. Palma M. S. Polybioside, a neuroactive compound from the venom of the social wasp Polybia Paulista. J Nat Prod, (2010), 73, 527–531. 20158240 10.1021/np900424t

[34] Shiraishi T Yokota S-i. Lactic acid Bacteria: Methods and protocols. New York, NY: Springer (2024).

[35] Chukiatsiri S Wongsrangsap N Kiatwuthinon P Phonphoem W. Purification and identification of novel antioxidant peptides derived from *Bombyx mori* pupae hydrolysates. Biochemistry and Biophysics Reports. (2024) 38:101707. doi: 10.1016/j.bbrep.2024.101707, PMID: 38601751 PMC11004502

[36] Ilyas M Rahman MU Ali M Deng T Farooqui NA Ali S. Effect of marine-derived scallop peptide hydrolysate on immune modulation and gut microbiota restoration in cyclophosphamide-induced immunosuppressed mice. Food Sci Nutr. (2025) 13:e70421. doi: 10.1002/fsn3.70421, PMID: 40703615 PMC12284433

[37] Alioui Y Ullah H Ali S Rahman MU Elkharti M Farooqui NA. Polysaccharides derived from golden mushroom (*Cantharellus cibarius* Fr.) modulate gut microbiota and enhance intestinal barrier function to ameliorate dextran sulfate sodium-induced colitis in mice. Front Pharmacol. (2024) 15:1498625. doi: 10.3389/fphar.2024.1498625, PMID: 39744127 PMC11688367

[38] Ali M Ullah H Farooqui NA Deng T Siddiqui NZ Ilyas M. NF-κB pathway activation by Octopus peptide hydrolysate ameliorates gut dysbiosis and enhances immune response in cyclophosphamide-induced mice. Heliyon. (2024) 10:e38370. doi: 10.1016/j.heliyon.2024.e38370, PMID: 39403534 PMC11472078

[39] Wang D Hu B Hu C Zhu F Liu X Zhang J. Clinical characteristics of 138 hospitalized patients with 2019 novel coronavirus-infected pneumonia in Wuhan, China. JAMA. (2020) 323:1061–9. doi: 10.1001/jama.2020.1585, PMID: 32031570 PMC7042881

[40] Grief SN Loza JK. Guidelines for the evaluation and treatment of pneumonia. Prim Care. (2018) 45:485–503. doi: 10.1016/j.pop.2018.04.001, PMID: 30115336 PMC7112285

[41] Fischer AH Jacobson KA Rose J Zeller R. Hematoxylin and eosin staining of tissue and cell sections. Cold Spring Harb Protoc. (2008) 2008:pdb.prot4986. doi: 10.1101/pdb.prot4986 21356829

[42] Xu K Guo Y Ping L Qiu Y Liu Q Li Z. Protective effects of SIRT6 overexpression against DSS-induced colitis in mice. Cells. (2020) 9:1513. doi: 10.3390/cells9061513, PMID: 32580272 PMC7348883

[43] Nunes NS Chandran P Sundby M Visioli F da Costa Gonçalves F Burks SR. Therapeutic ultrasound attenuates DSS-induced colitis through the cholinergic anti-inflammatory pathway. EBioMedicine. (2019) 45:495–510. doi: 10.1016/j.ebiom.2019.06.033, PMID: 31253515 PMC6642284

[44] Yang J Lin J Gu T Sun Q Xu W Peng Y. Chicoric acid effectively mitigated dextran sulfate sodium (DSS)-induced colitis in BALB/c mice by modulating the gut microbiota and fecal metabolites. Int J Mol Sci. (2024) 25:841. doi: 10.3390/ijms25020841, PMID: 38255916 PMC10815209

[45] Silva IAN Gvazava N Bölükbas DA Stenlo M Dong J Hyllen S. A semi-quantitative scoring system for green histopathological evaluation of large animal models of acute lung injury. Bio Protoc. (2022) 12:4493. doi: 10.21769/BioProtoc.4493, PMID: 36199700 PMC9486691

[46] Troeger C Blacker B Khalil IA Rao PC Cao J Zimsen SR. Estimates of the global, regional, and national morbidity, mortality, and aetiologies of lower respiratory infections in 195 countries, 1990–2016: a systematic analysis for the global burden of disease study 2016. Lancet Infect Dis. (2018) 18:1191–210. doi: 10.1016/S1473-3099(18)30310-4, PMID: 30243584 PMC6202443

[47] World Health Organization. Tracking universal health coverage: 2023 global monitoring report. Geneva: World Health Organization (2023).

[48] Muszyńska B Grzywacz-Kisielewska A Kała K Gdula-Argasińska J. Anti-inflammatory properties of edible mushrooms: a review. Food Chem. (2018) 243:373–81. doi: 10.1016/j.foodchem.2017.09.149, PMID: 29146352

[49] Miao X-P Sun X-N Cui L-J Cao Q-F Zhuang G-F Deng T-Z. Suppressive effect of pectic polysaccharides extracted from Rauwolfia verticillata (Lour.) Baill. var. hainanensis Tsiang on inflammation by regulation of NF–κ B pathway and interleukin–17 in mice with dextran sulphatesodium–induced ulcerative colitis. Asian Pac J Trop Med. (2015) 8:147–52. doi: 10.1016/S1995-7645(14)60306-0, PMID: 25902030

[50] Kim Y Sung J Sung M Choi Y Jeong H-S Lee J. Involvement of heme oxygenase-1 in the anti-inflammatory activity of Chrysanthemum boreale Makino extracts on the expression of inducible nitric oxide synthase in RAW264. 7 macrophages. J Ethnopharmacol. (2010) 131:550–4. doi: 10.1016/j.jep.2010.07.030, PMID: 20656003

[51] Najafian L Babji A. A review of fish-derived antioxidant and antimicrobial peptides: their production, assessment, and applications. Peptides. (2012) 33:178–85. doi: 10.1016/j.peptides.2011.11.013, PMID: 22138166

[52] Khan AI Rehman AU Farooqui NA Siddiqui NZ Ayub Q Ramzan MN. Shrimp peptide hydrolysate modulates the immune response in cyclophosphamide immunosuppressed mice model. J Food Biochem. (2022) 46:e14251. doi: 10.1111/jfbc.14251 35633198

[53] Barenholz-Paniry V Ishay JS Pick IA Hammel I. Mast cell activation by hornet (*Vespa orientalis*) venom. Int Arch Allergy Immunol. (1990) 93:178–83. doi: 10.1159/000235298, PMID: 2099343

[54] Jin C-l Zhang Z-m Ye J-l Gao C-q Yan H-c Li H-c. Lysine-induced swine satellite cell migration is mediated by the FAK pathway. Food Funct. (2019) 10:583–91. doi: 10.1039/c8fo02066c, PMID: 30672919

[55] Zhao H Raines LN Huang SC-C. Carbohydrate and amino acid metabolism as hallmarks for innate immune cell activation and function. Cells. (2020) 9:562. doi: 10.3390/cells9030562, PMID: 32121028 PMC7140477

[56] Qi M Wang J Li J Liao S Liu Y Yin Y. Dietary glutamine, glutamate, and aspartate supplementation improves hepatic lipid metabolism in post-weaning piglets. Anim Nutr. (2020) 6:124–9. doi: 10.1016/j.aninu.2019.12.002, PMID: 32542191 PMC7283369

[57] Stachowicz K Bobula B Kusek M Lenda T Tokarski K. Evidence for the interaction of COX-2 with mGluR5 in the regulation of EAAT1 and EAAT3 protein levels in the mouse hippocampus. The influence of oxidative stress mechanisms. Brain Res. (2021) 1771:147660. doi: 10.1016/j.brainres.2021.147660, PMID: 34529964

[58] Kelly B Pearce EL. Amino assets: how amino acids support immunity. Cell Metab. (2020) 32:154–75. doi: 10.1016/j.cmet.2020.06.010, PMID: 32649859

[59] Wang C Liu Z Zhou T Wu J Feng F Wang S. Gut microbiota-derived butyric acid regulates calcific aortic valve disease pathogenesis by modulating GAPDH lactylation and butyrylation. iMeta. (2025) 4:e70048. doi: 10.1002/imt2.70048, PMID: 40860435 PMC12371252

[60] Friedrich M Pohin M Powrie F. Cytokine networks in the pathophysiology of inflammatory bowel disease. Immunity. (2019) 50:992–1006. doi: 10.1016/j.immuni.2019.03.017, PMID: 30995511

[61] Zadeh M Khan MW Goh YJ Selle K Owen JL Klaenhammer T. Induction of intestinal pro-inflammatory immune responses by lipoteichoic acid. J Inflamm. (2012) 9:1–12. doi: 10.1186/1476-9255-9-7, PMID: 22423982 PMC3325164

[62] Wang K Jin X Chen Y Song Z Jiang X Hu F. Polyphenol-rich propolis extracts strengthen intestinal barrier function by activating AMPK and ERK signaling. Nutrients. (2016) 8:272. doi: 10.3390/nu8050272, PMID: 27164138 PMC4882685

[63] Stolfi C Maresca C Monteleone G Laudisi F. Implication of intestinal barrier dysfunction in gut dysbiosis and diseases. Biomedicine. (2022) 10:289. doi: 10.3390/biomedicines10020289, PMID: 35203499 PMC8869546

[64] Di Tommaso N Gasbarrini A Ponziani FR. Intestinal barrier in human health and disease. Int J Environ Res Public Health. (2021) 18:12836. doi: 10.3390/ijerph182312836, PMID: 34886561 PMC8657205

[65] Xu Y Feng H Zhang Z Zhang Q Tang J Zhou J. The protective role of *scorias spongiosa* polysaccharide-based microcapsules on intestinal barrier integrity in dss-induced colitis in mice. Foods. (2023) 12:669. doi: 10.3390/foods12030669, PMID: 36766197 PMC9914818

[66] Kubysheva N Boldina M Eliseeva T Soodaeva S Klimanov I Khaletskaya A. Relationship of serum levels of IL-17, IL-18, TNF-α, and lung function parameters in patients with COPD, asthma-COPD overlap, and bronchial asthma. Mediat Inflamm. (2020) 2020:1–11. doi: 10.1155/2020/4652898, PMID: 32733164 PMC7372292

[67] Al-Qahtani AA Alhamlan FS Al-Qahtani AA. Pro-inflammatory and anti-inflammatory interleukins in infectious diseases: a comprehensive review. Tropical medicine and infectious disease. (2024) 9:13. doi: 10.3390/tropicalmed9010013, PMID: 38251210 PMC10818686

[68] Ullah H Deng T Ali M Farooqui NA Alsholi DM Siddiqui NZ. Sea conch peptides hydrolysate alleviates DSS-induced colitis in mice through immune modulation and gut microbiota restoration. Molecules. (2023) 28:6849. doi: 10.3390/molecules28196849, PMID: 37836692 PMC10574497

[69] Zhang T Ma C Zhang Z Zhang H Hu H. NF-κB signaling in inflammation and cancer. MedComm. (2021) 2:618–53. doi: 10.1002/mco2.104, PMID: 34977871 PMC8706767

[70] Ma Q Hao S Hong W Tergaonkar V Sethi G Tian Y. Versatile function of NF-ĸB in inflammation and cancer. Exp Hematol Oncol. (2024) 13:68. doi: 10.1186/s40164-024-00529-z, PMID: 39014491 PMC11251119

[71] Xu A Deng F Chen Y Kong Y Pan L Liao Q. NF-κB pathway activation during endothelial-to-mesenchymal transition in a rat model of doxorubicin-induced cardiotoxicity. Biomed Pharmacother. (2020) 130:110525. doi: 10.1016/j.biopha.2020.110525, PMID: 32702633

[72] Karin M Greten FR. NF-κB: linking inflammation and immunity to cancer development and progression. Nat Rev Immunol. (2005) 5:749–59. doi: 10.1038/nri1703, PMID: 16175180

[73] Park MH Hong JT. Roles of NF-κB in cancer and inflammatory diseases and their therapeutic approaches. Cells. (2016) 5:15. doi: 10.3390/cells5020015, PMID: 27043634 PMC4931664

[74] Takeuchi O Akira S. Pattern recognition receptors and inflammation. Cell. (2010) 140:805–20. doi: 10.1016/j.cell.2010.01.022, PMID: 20303872

[75] Quinton LJ Jones MR Simms BT Kogan MS Robson BE Skerrett SJ. Functions and regulation of NF-κB RelA during pneumococcal pneumonia. J Immunol. (2007) 178:1896–903. doi: 10.4049/jimmunol.178.3.1896, PMID: 17237440 PMC2674289

[76] Liu S Gao J Zhu M Liu K Zhang H-L. Gut microbiota and dysbiosis in Alzheimer’s disease: implications for pathogenesis and treatment. Mol Neurobiol. (2020) 57:5026–43. doi: 10.1007/s12035-020-02073-3, PMID: 32829453 PMC7541367

[77] Lau WL Tran T Rhee CM Kalantar-Zadeh K Vaziri ND. Diabetes and the gut microbiome. Semin Nephrol. (2021) 41:104–13. doi: 10.1016/j.semnephrol.2021.03.005 34140089

[78] Bai X Wei H Liu W Coker OO Gou H Liu C. Cigarette smoke promotes colorectal cancer through modulation of gut microbiota and related metabolites. Gut. (2022) 71:2439–50. doi: 10.1136/gutjnl-2021-325021, PMID: 35387878 PMC9664112

[79] Chidambaram SB Essa MM Rathipriya A Bishir M Ray B Mahalakshmi AM. Gut dysbiosis, defective autophagy and altered immune responses in neurodegenerative diseases: Tales of a vicious cycle. Pharmacol Ther. (2022) 231:107988. doi: 10.1016/j.pharmthera.2021.107988, PMID: 34536490

[80] Li H Wang Z Guan Z Miao J Li W Yu P. Ucfnnet: ulcerative colitis evaluation based on fine-grained lesion learner and noise suppression gating. Comput Methods Prog Biomed. (2024) 247:108080. doi: 10.1016/j.cmpb.2024.108080 38382306

[81] Zhuang H Cheng L Wang Y Zhang Y-K Zhao M-F Liang G-D. Dysbiosis of the gut microbiome in lung cancer. Front Cell Infect Microbiol. (2019) 9:112. doi: 10.3389/fcimb.2019.00112, PMID: 31065547 PMC6489541

[82] Zhao J Zhang X Liu H Brown MA Qiao S. Dietary protein and gut microbiota composition and function. Current Protein and Peptide Science. (2019) 20:145–54. doi: 10.2174/1389203719666180514145437, PMID: 29756574

[83] Sencio V Machado MG Trottein F. The lung–gut axis during viral respiratory infections: the impact of gut dysbiosis on secondary disease outcomes. Mucosal Immunol. (2021) 14:296–304. doi: 10.1038/s41385-020-00361-8, PMID: 33500564 PMC7835650

[84] Whiteside SA McGinniss JE Collman RG. The lung microbiome: progress and promise. J Clin Invest. (2021) 131:473. doi: 10.1172/JCI150473, PMID: 34338230 PMC8321564

[85] Elgamal Z Singh P Geraghty P. The upper airway microbiota, environmental exposures, inflammation, and disease. Medicina. (2021) 57:823. doi: 10.3390/medicina57080823, PMID: 34441029 PMC8402057

[86] Willers M Viemann D. Role of the gut microbiota in airway immunity and host defense against respiratory infections. Biol Chem. (2021) 402:1481–91. doi: 10.1515/hsz-2021-0281, PMID: 34599869

[87] Acevedo-Román A Pagán-Zayas N Velázquez-Rivera LI Torres-Ventura AC Godoy-Vitorino F. Insights into gut dysbiosis: inflammatory diseases, obesity, and restoration approaches. Int J Mol Sci. (2024) 25:9715. doi: 10.3390/ijms25179715, PMID: 39273662 PMC11396321

[88] Wen Y Ullah H Ma R Farooqui NA Li J Alioui Y. Anemarrhena asphodeloides Bunge polysaccharides alleviate lipoteichoic acid-induced lung inflammation and modulate gut microbiota in mice. Heliyon. (2024) 10:e39390. doi: 10.1016/j.heliyon.2024.e39390, PMID: 39469699 PMC11513480

[89] Kirsch M. Bacterial overgrowth. Am J Gastroenterol. (1990) 1:85 2178395

[90] Sorathia S. J. Chippa V. Rivas J. M. Small intestinal bacterial overgrowth, Treasure Island, Florida, USA: StatPearls Publishing. (2019). 31536241

[91] Du Y He C An Y Huang Y Zhang H Fu W. The role of short chain fatty acids in inflammation and body health. Int J Mol Sci. (2024) 25:7379. doi: 10.3390/ijms25137379, PMID: 39000498 PMC11242198

[92] Montgomery TL Toppen LC Eckstrom K Heney ER Kennedy JJ Scarborough MJ. Lactobacillaceae differentially impact butyrate-producing gut microbiota to drive CNS autoimmunity. Gut Microbes. (2024) 16:2418415. doi: 10.1080/19490976.2024.2418415, PMID: 39462277 PMC11520542

[93] Bor B Poweleit N Bois JS Cen L Bedree JK Zhou ZH. Phenotypic and physiological characterization of the epibiotic interaction between TM7x and its basibiont *Actinomyces*. Microb Ecol. (2016) 71:243–55. doi: 10.1007/s00248-015-0711-7, PMID: 26597961 PMC4688200

[94] Lu K Li C Men J Xu B Chen Y Yan P. Traditional Chinese medicine to improve immune imbalance of asthma: focus on the adjustment of gut microbiota. Front Microbiol. (2024) 15:1409128. doi: 10.3389/fmicb.2024.1409128, PMID: 39411430 PMC11473343

[95] Li L-Q Chen X Zhu J Zhang S Chen S-Q Liu X. Advances and challenges in interaction between heteroglycans and *Bifidobacterium*: utilization strategies, intestinal health and future perspectives. Trends Food Sci Technol. (2023) 134:112–22. doi: 10.1016/j.tifs.2023.02.018

[96] Muhlebach MS Hatch JE Einarsson GG McGrath SJ Gilipin DF Lavelle G. Anaerobic bacteria cultured from cystic fibrosis airways correlate to milder disease: a multisite study. Eur Respir J. (2018) 52:2018. doi: 10.1183/13993003.00242-2018, PMID: 29946004 PMC6376871

