# Supplementary material for: Vespa orientalis pupae peptide hydrolysates modulate NF-κB signaling in LTA-induced pneumonia from clinical Enterococcus faecalis isolates: implications for gut microbiota
Source: Front Nutr. 2025 Sep 23;12:1651499. doi: 10.3389/fnut.2025.1651499 (PMC12500668; doi:10.3389/fnut.2025.1651499)
Supplement: Supplementary file 1 [file Data_Sheet_1.docx]

**Supplementary Figures & Tables**


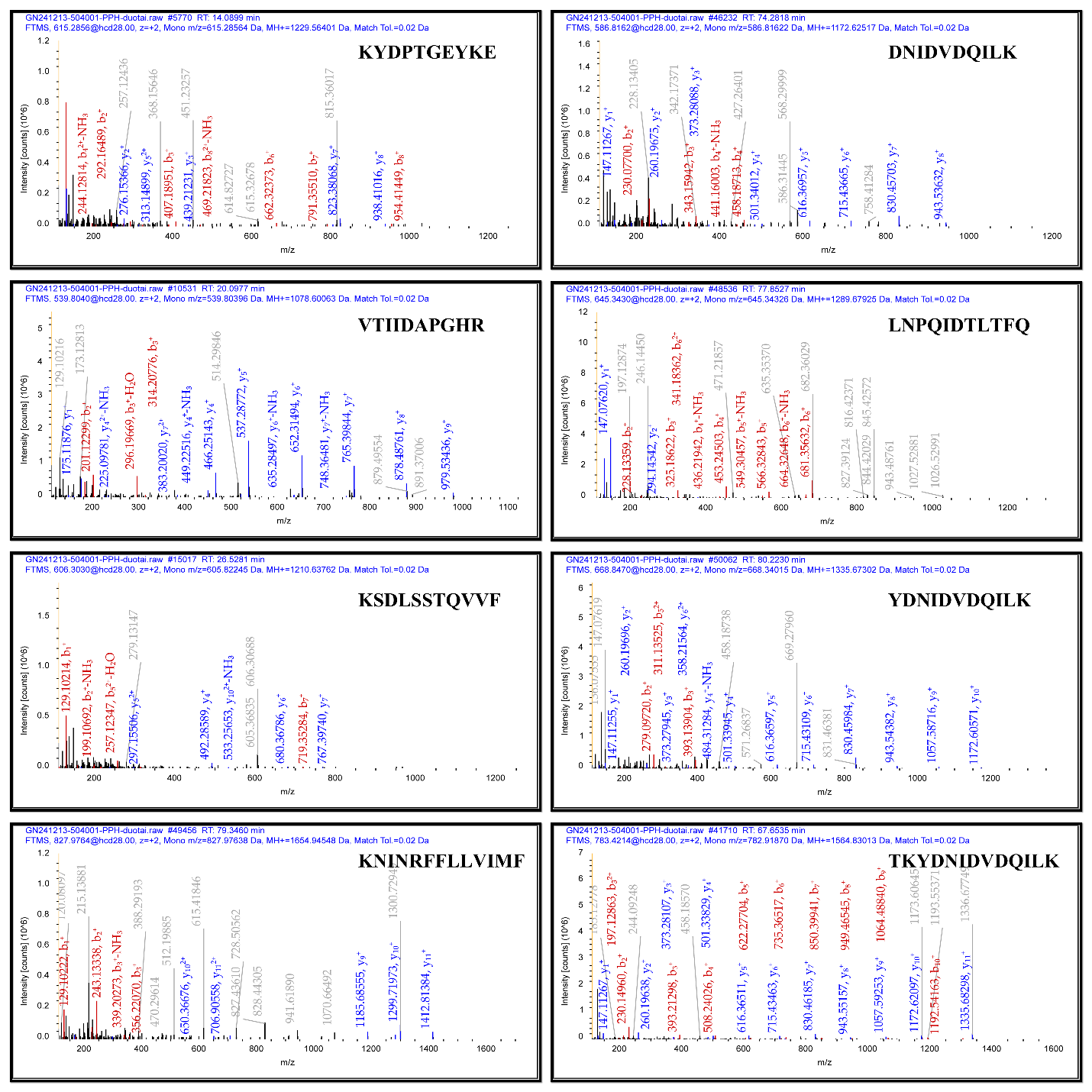


**Figure 1** MALDI-TOF-MS analysis of peptides from *Vespa orientalis* pupae peptide hydrolysate. Representing peptides with unique molecular masses and varying amino acid compositions, indicating the heterogeneous nature of the hydrolysate.

Table 1. The concentrations and composition of different amino acids in pupae peptide hydrolysate.

| S. No | Amino acid type | Concentration g/100g |
| --- | --- | --- |
| 1 | Glutamic acid (Glu) | 10.3 |
| 2 | Aspartic acid (Asp) | 5.42 |
| 3 | Leucine (Leu) | 3.86 |
| 4 | Valine (Val), | 3.56 |
| 5 | Glycine (Gly) | 3.47 |
| 6 | Lysine (Lys) | 3.33 |
| 7 | Tyrosine (Tyr) | 2.77 |
| 8 | Alanine (Ala) | 2.64 |
| 9 | Proline (Pro) | 2.62 |
| 10 | Isoleucine (IIe) | 2.32 |
| 11 | Histidine (His) | 2.31 |
| 12 | Arginine (Arg) | 2.12 |
| 13 | Threonine (Thr) | 2.02 |
| 14 | Serine (Ser) | 1.60 |
| 15 | Phenylalanine (Phe) | 1.27 |
| 16 | Methionine (Met) | 0.56 |
| 17 | Cystine (Cys) | 0.14 |


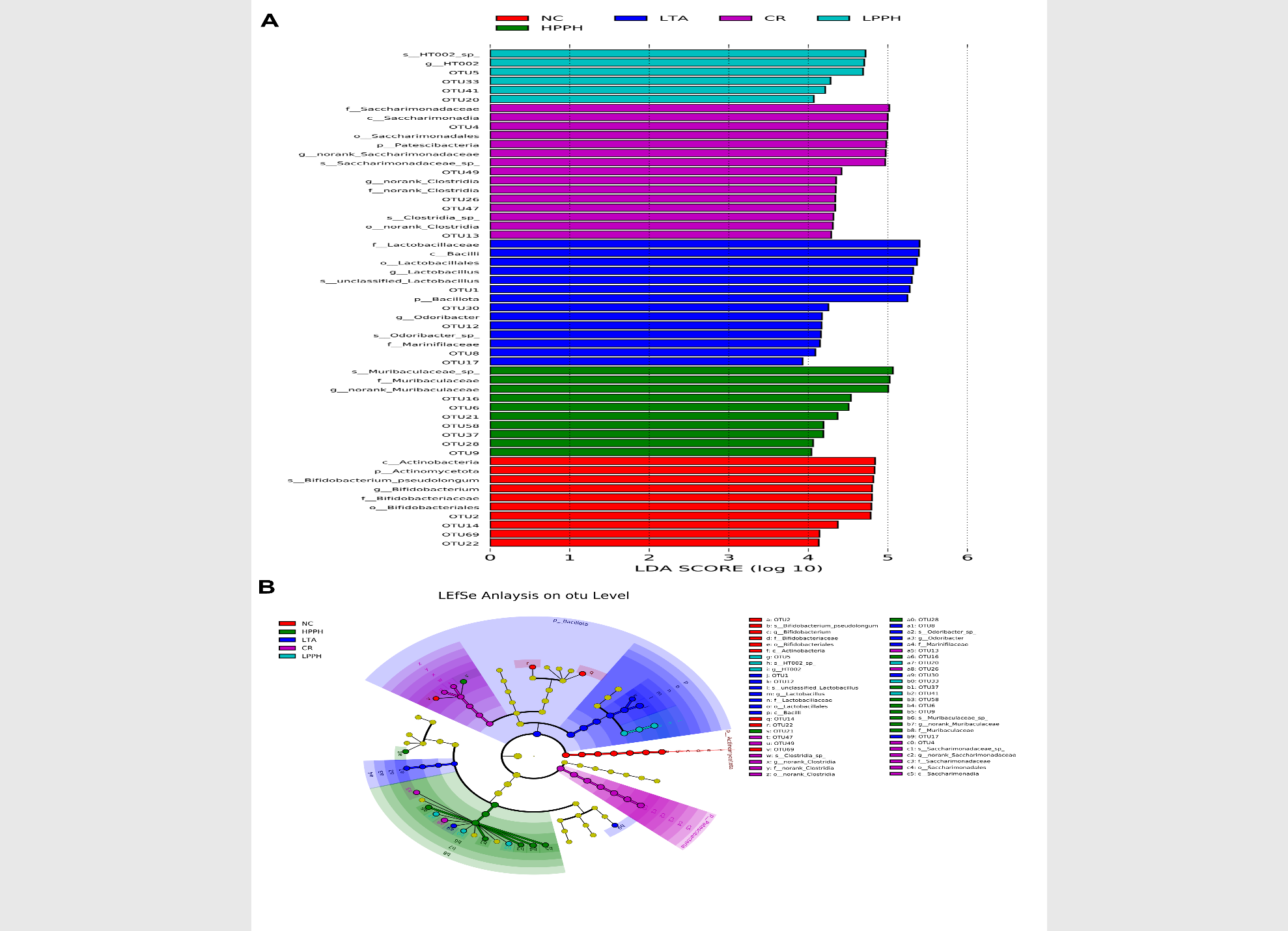


Figure 2. Presents the Linear Discriminant Analysis (LDA) effect size and cladogram, illustrating the variations in gut microbiota among the experimental groups. Panel (A) features the LDA bar chart, which identifies taxa with significantly different abundance levels (LDA score) across five groups: NC (normal control), LTA (Disease Model), LPPH (low-dose PPH, 200 mg/kg), HPPH (high-dose PPH, 400 mg/kg), and CR (crude extract, 300 mg/kg). Positive LDA scores indicate enrichment in the respective group. Panel (B) displays the LEfSe cladogram, depicting the phylogenetic relationships of taxa that are significantly enriched. Nodes are colored according to the group in which they are most prevalent. Taxa enriched in HPPH include Bifidobacterium pseudolongum, Bifidobacterium, and Lactobacillus spp., while LTA is characterized by Clostridia_UCG_014. The NC group shows enrichment in Muribaculaceae, the LPPH group in Odoribacter and Marinifilaceae, and the CR group in Candidatus Saccharimonas.


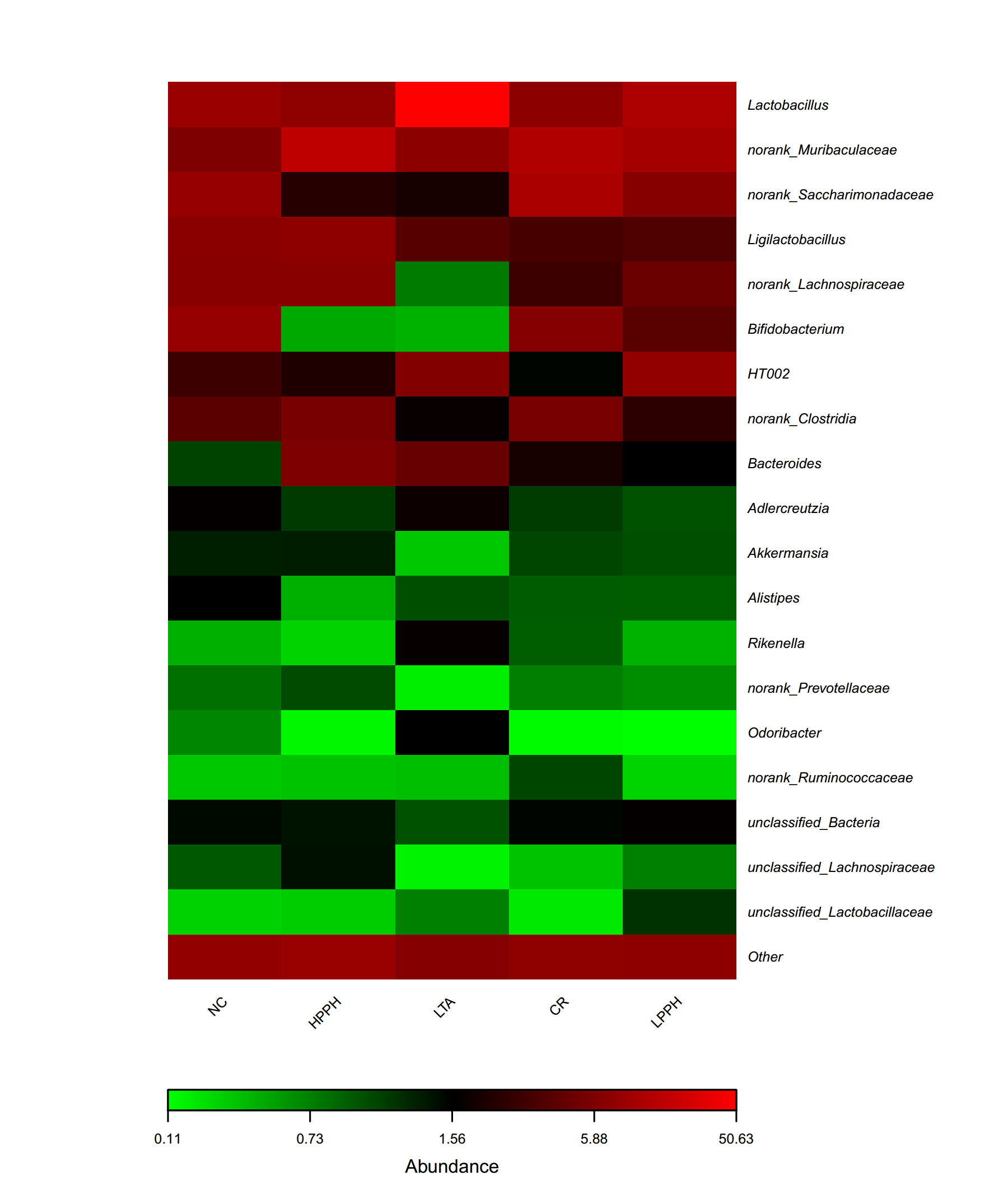


Figure 3. Heatmap depicting the taxonomic abundance at genus level of bacterial communities across various sample groups. This heatmap illustrates the relative abundance of bacterial taxa among different groups (NC, LTA, LPPH, HPPH, and CR). The color intensity represents abundance levels, ranging from 0.11 (light green, indicating low abundance) to 50.63 (dark red, indicating high abundance). Key bacterial groups include Lactobacillus, Muribaculaceae, Saccharimonadaceae, Bifidobacterium, and several unclassified taxa. The analysis reveals distinct clustering patterns of microbial communities, with certain taxa demonstrating a preference for specific sample conditions.
